# Jasmonic acid and ERF family genes are involved in chilling sensitivity and seed browning of pepper fruit after harvest

**DOI:** 10.1038/s41598-020-75055-z

**Published:** 2020-10-21

**Authors:** Jeong Gu Lee, Gibum Yi, Jieun Seo, Byoung-Cheorl Kang, Jeong Hee Choi, Eun Jin Lee

**Affiliations:** 1 Depatment of Agriculture, Forestry and Bioresources, College of Agriculture and Life Sciences, Seoul National University, Seoul, 08826 Republic of Korea; 2 Plant Genomics and Breeding Institute, Seoul National University, Seoul, 08826 Republic of Korea; 3 Korea Food Research Institute, Wanju-gun, Jeollabuk-do 55365 Republic of Korea; 4 Research Institute of Agriculture and Life Sciences, Seoul National University, Seoul, 08826 Republic of Korea

**Keywords:** Molecular biology, Plant sciences

## Abstract

Pepper (*Capsicum annuum* L.) fruit is sensitive to temperatures below 10 °C, which severely diminish fruit quality during cold chain distribution. Seed browning was a major chilling symptom in 36 genotypes of *C. annuum* fruit screened after storage at 2 °C for 3 weeks. Among them, pepper fruits of chilling-insensitive ‘*UZB-GJG-1999–51*’ and -sensitive ‘*C00562*’ were treated at 2 °C for 0 or 24 h, respectively. Analyses of integrated transcriptome-metabolome and relative gene expression in seeds obtained from the two genotypes were conducted to identify key factors involved in the seed browning induced by chilling. The relative contents of branched-chain amino acids such as leucine, isoleucine, and valine were significantly increased after chilling. Transcriptome identification showed 3,140 differentially expressed genes (log twofold change > 1.0 and FDR-corrected *p* value < 0.05) affected by chilling between the two genotypes. Particularly, genes related to jasmonic acid synthesis and signaling were differentially expressed. A regulatory network of jasmonic acid synthesis and signaling, and regulation of ERF family genes might contribute to chilling response in pepper fruit. The results of this study may help facilitate further studies to develop chilling-insensitive peppers and could be a basis for improving postharvest fruit quality.

## Introduction

Harvested crops are usually stored at low temperatures during the distribution and sale processes to maintain their freshness. However, pepper (*Capsicum annuum* L.) fruit, a subtropical crop, is sensitive to low temperatures. Various chilling symptoms appear at storage temperatures below 7–10 °C depending on the variety, fruit maturity, and time of exposure to chilling temperatures^1^. Typical chilling symptoms in pepper fruit are; seed browning or discoloration, pitting on the surface, calyx discoloration, and shrinkage^2^. These injuries lower the marketability of pepper fruit during cold chain distribution.

Chilling symptoms have been known to result from the deterioration of cellular membranes by reactive oxygen species (ROS), mostly derived from the lipid peroxidation process^3^. In addition, jasmonic acid (JA) and salicylic acid (SA)-synthesis and -signaling pathways have been known to be involved in protecting against chilling stress in plants^4,5^. JA is involved in the chilling stress mechanism and it regulates chilling tolerance^6,7^. An increase in endogenous JA content under chilling stress in Arabidopsis^8^, tomato^9^, and pepper fruit^5^ has been reported. JA is synthesized from α-linolenic acid and lipoxygenase (LOX), allene oxide synthase (AOS), and allene oxide cyclase (AOC), which are enzymes involved in JA biosynthesis^10^. In order for JA to have biological activity, it conjugates with isoleucine to form jasmonoyl-isoleucine (JA-Ile), a JA active derivative; jasmonate resistant 1 (JAR1) is involved to form JA-Ile.

In the JA signaling pathway, one of the most important proteins is JASMONAT-ZIM-domain (JAZ), which acts as a repressor for JA signaling. If JA is absent or present in low levels, JAZ protein binds to downstream transcription factors such as MYC^11^, DELLA^12^, and the inducer of C-repeat binding factor expression (ICE)^8^. JAZ protein limits the transcription factor’s activities; however, in the presence of JA-Ile, JAZ protein is broken down by 26S proteasome, and then transcription factors for the expression of downstream genes that are needed for the stress response pathway are activated^13^. CORONATINE INSENSITIVE 1 (COI1), which belongs to the group of F-box proteins, binds to JAZ protein. Further, JA-Ile can bind to the COI1-JAZ complex to promote ubiquitination and degradation of JAZ protein. JA-Ile has been known to be required for COI1-mediated degradation of JAZ protein^14^.

Activation of apetala2/ethylene responsive factors (AP2/ERFs) such as ERF1^15^, ERF32^16^, and ORA59^17^ could begin in the presence of JA as well as under JAZ degradation. AP2/ERFs consist of two subfamilies: the inducer of the C-repeat binding factor/dehydration responsive element binding factor (CBF/DREB) and the ERF subfamily binding to GCC-box; both are involved in JA, SA, and ethylene responses to environmental stress. AP2/ERFs are important regulators for defense against abiotic stresses^18^. In particular, AP2/ERFs are known to be involved in cold stress in plants, and it has been reported that AP2/ERFs play a major role in chilling tolerance and cold acclimation in *Arabidopsis*^19^. However, the regulatory mechanism of AP2/ERFs in cold stress response is still unclear. In a recent study, it was reported that AP2/ERFs regulate the peroxidase-encoding genes^20^ WRKY33^21^ and CBF/DREB^22^, and mitigate reactive oxygen in response to cold stress.

The CBF/DREB subfamily binds to the dehydration-responsive element/C-repeat (DRE/CRT) elements and is regulated by the ICE transcription factor. As mentioned earlier, JAZ protein is known as a regulator of chilling stress because it suppresses the ICE transcription factor that is responsible for the chilling stress response. However, at chilling temperatures, an increased JA-Ile level activates ICE by the degradation of the COI1-JAZ complex^23^. Then, ICE regulates CBF/DREB to contribute to chilling tolerance through CBF regulons such as responsiveness to desiccation genes and cold-responsive genes^24^. Therefore, many members of the CBF/DREB subfamily are involved in improving the stress tolerance of various plants under the environmental stresses of cold, drought, and salinity.

To understand the underlying mechanism of seed browning induced by chilling, we first screened seed browning occurrences of 36 genotypes of *C. annuum* fruit. We also investigated the metabolomics and transcriptomics of two pepper genotypes that show great differences in their occurrence rates of seed browning as a chilling symptom when exposed to chilling temperatures after harvest. Our results showed JA synthesis, JA signaling, and ERF family genes contribute to chilling response in pepper fruit. This study can help elucidate the cellular mechanism or identify key factors affecting chilling sensitivity or insensitivity of peppers after harvest. Also, this study provides information for further studies to develop chilling-insensitive pepper fruit and could be a basis for improving the postharvest fruit quality of peppers.

## Results

### Seed browning rates of 36 pepper genotypes

Seed browning symptoms were the major chilling injury in the 36 genotypes of *C. annuum* screened in this study (Supplementary Table S1). Other chilling symptoms such as pitting on the surface and discoloration of the calyx were negligible during cold storage at 2 °C for 3 weeks. Seed browning was also observed as a major chilling symptom of *C. annuum* ‘Cheongyang’ fruit treated at 2 °C for 3 weeks^5^. We sorted 36 pepper genotypes according to seed browning rates from 0.0% (chilling-insensitive peppers) to over 60.0% (chilling-sensitive peppers) (Supplementary Table S1).

**Figure 1:**
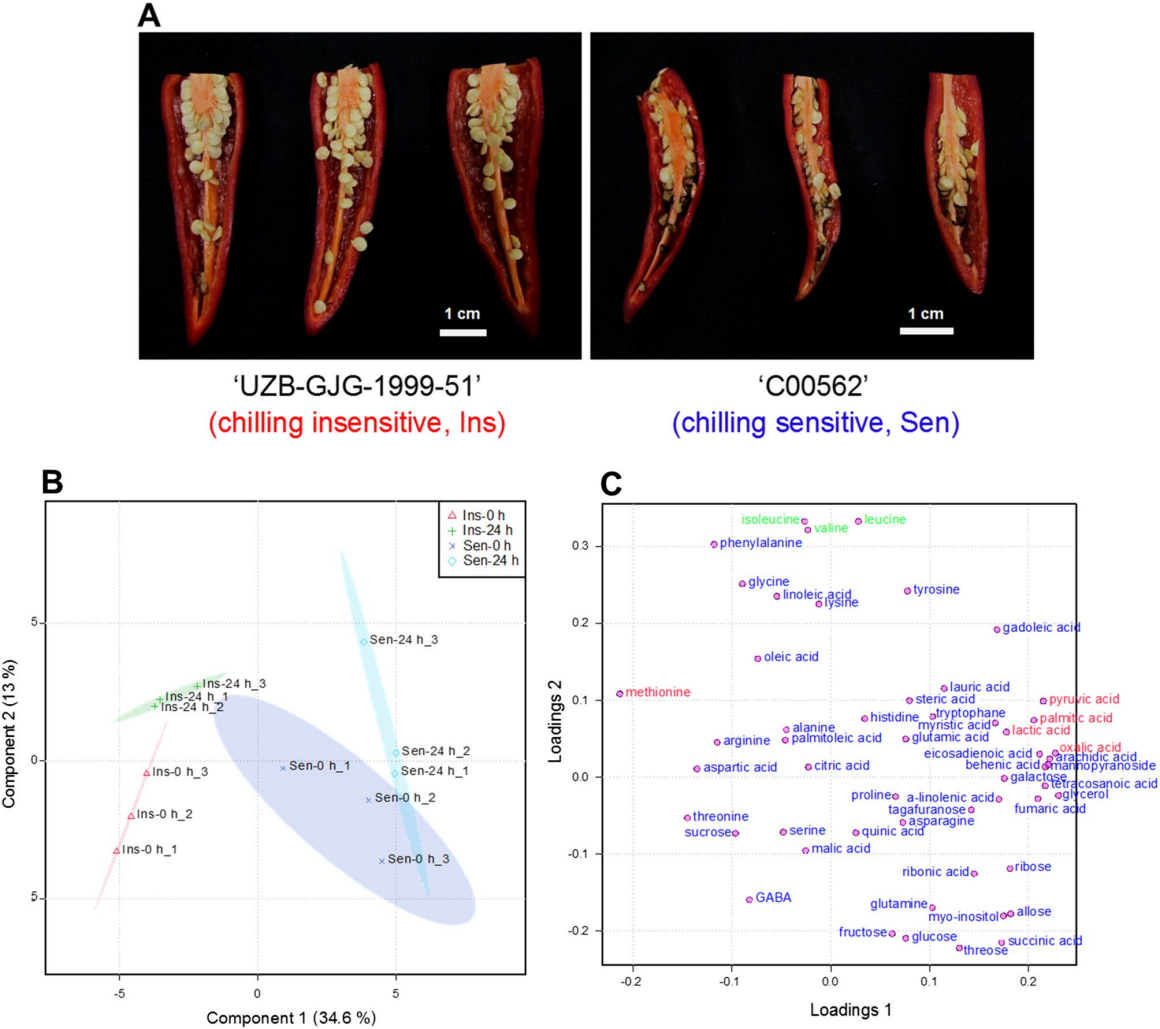
Seed browning and metabolite analysis (**A**–**C**). Seed browning appearances (**A**) of ‘*UZB-GJG-1999-51*’ (chilling-insensitive, Ins) and ‘*C00562*’ (chilling-sensitive, Sen) peppers induced by cold storage at 2 °C for 3 weeks. Analysis of PLS-DA score plot (**B**) and loading plot (**C**) of metabolites in seeds obtained from both peppers after chilling treatment at 2 °C for 0 h and 24 h, respectively. (**B**, **C**) were created with freely available MetaboAnalyst (version 3.0, https://www.metaboanalyst.ca/).

During cold storage at 2 °C for 3 weeks, there were great differences in seed browning rates between the chilling-insensitive ‘*UZB-GJG-1999-51*’ and chilling-sensitive ‘*C00562*’ (Fig. 1A and Supplementary Table S1). The seed browning rate of chilling-sensitive ‘*C00562*’ was 63.86%, whereas the seed browning rate of chilling-insensitive ‘*UZB-GJG-1999-51*’ was 0.00%. Additionally, the seed browning rates of other chilling-insensitive genotypes ‘Takanotsume’ and ‘Hungarian Wax’, and other chilling-sensitive genotypes ‘Chili bangi’ and ‘Gyeonggiyangpyeong’ were in the order of 0.00%, 4.31%, 30.88%, and 41.01%, respectively (Supplementary Fig. S1 and Supplementary Table S1).

### Differentially accumulated metabolites

A total 51 of untargeted and targeted metabolites in pepper seeds were quantified in this study. To confirm the relationships among identified metabolites, partial least squares-discriminant analysis (PLS-DA) was performed (Fig. 1B,C). The first and second principle components (PCs) accounted for 47.6% (PC 1, 34.6%; PC 2, 13.0%) of the variance in the dataset. The PLS-DA loading plot (Fig. 1C) was used to identify the causative metabolites of the PLS-DA score plot. We were able to confirm that pyruvic acid, lactic acid, oxalic acid, palmitic acid, and methionine were the major metabolites separating the two pepper genotypes (chilling-insensitive ‘*UZB-GJG-1999-51*’ and chilling-sensitive ‘*C00562*’). In addition, among amino acids, isoleucine, valine, and leucine were the major metabolites contributing to the distribution between ‘0 h’ and ‘24 h’ pepper groups, with separation due to the chilling treatment at 2 °C for 24 h.

**Figure 2:**
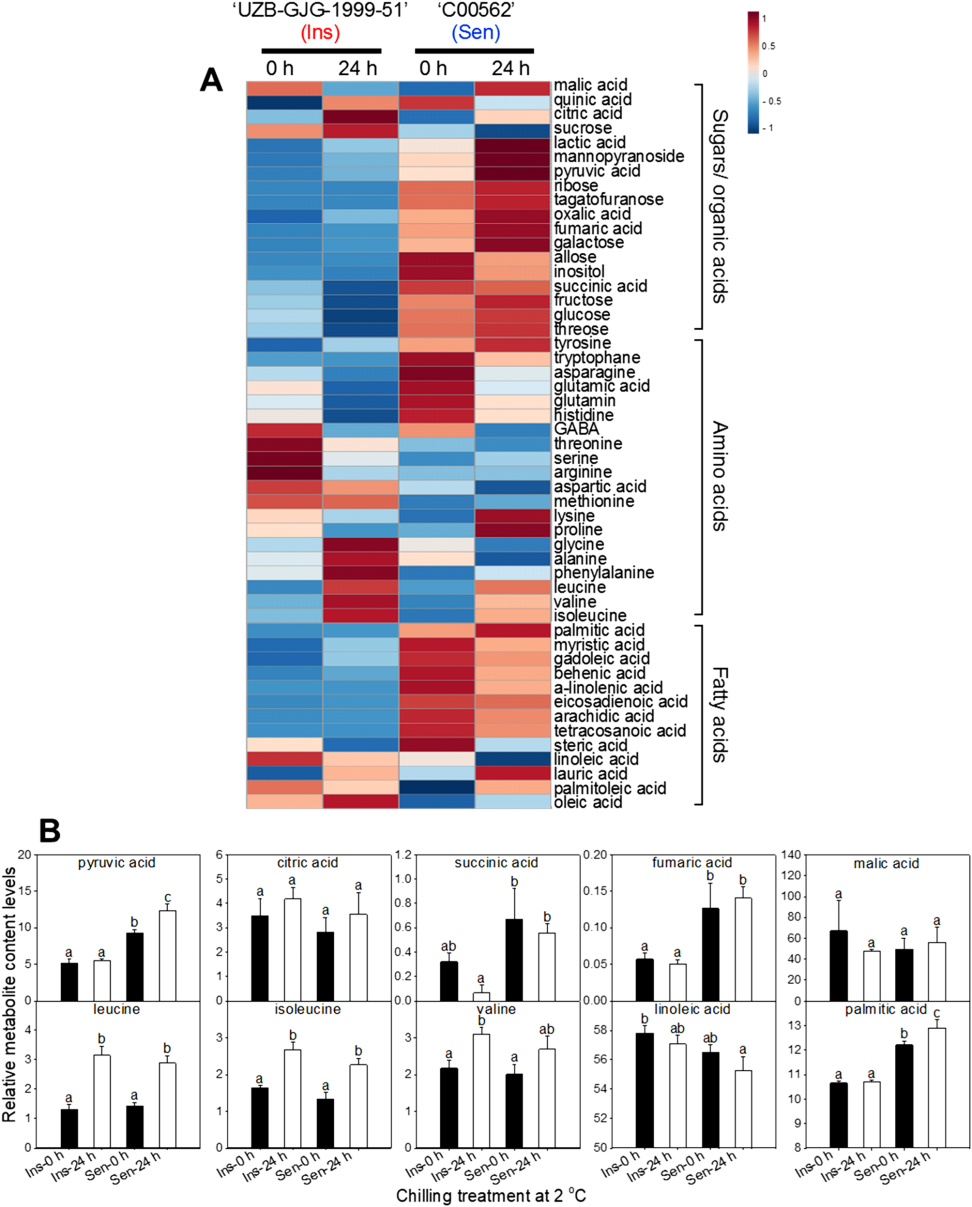
Differentially accumulated metabolites (DAMs) (**A**, **B**). Heat-map analysis (**A**) of DAMs and the relative metabolite levels (**B**) of 10 selected organic acids, amino acids, and fatty acids of seeds obtained from ‘*UZB-GJG-1999-51*’ (chilling-insensitive, Ins)’ and ‘*C00562*’ (chilling-sensitive, Sen) peppers after chilling treatment at 2 °C for 0 h and 24 h, respectively. Vertical bars are means ± SD (n = 3). Different letters represent significant differences (*p* < 0.05) in Duncan’s multiple range test. (**A**) was created with freely available MetaboAnalyst (version 3.0, https://www.metaboanalyst.ca/).

Figure 2A shows the heat-map analysis of metabolites. Most organic acids, except for malic acid and citric acid, showed substantially higher contents in chilling-sensitive ‘*C00562*’ than in chilling-insensitive ‘*UZB-GJG-1999-51*′. Also, leucine, valine, and isoleucine contents were clearly increased in the two pepper genotypes by chilling treatment; however, chilling-insensitive ‘*UZB-GJG-1999-51*′ had substantially higher contents of leucine, valine, and isoleucine than chilling-sensitive ‘*C00562*’.

The relative content levels of 10 selected metabolites from organic acids, amino acids, and fatty acids are compared in Fig. 2B. The levels of pyruvic acid, succinic acid, and fumaric acid were substantially higher in chilling-sensitive ‘*C00562*’, regardless of chilling treatment, at 2 °C for 24 h. The pyruvic acid level was significantly increased in ‘*C00562*’ only after chilling treatment. In the cases of leucine, isoleucine, and valine, which are branched-chain amino acids (BCAA), there were no differences in their contents between the two pepper genotypes, and they showed similar levels to each other at 0 h, before chilling treatment. However, after chilling treatment (24 h), the three amino acid levels were significantly increased in the two pepper genotypes.

The level of palmitic acid was not significantly changed in chilling-insensitive ‘*UZB-GJG-1999-51*’ by chilling treatment, but it was significantly increased in chilling-sensitive ‘*C00562*’. Citric acid and malic acid levels were not significantly different. Linoleic acid content was slightly higher in chilling-insensitive ‘*UZB-GJG-1999-51*’ than in chilling-sensitive ‘*C00562*’ but there were no significant differences according to chilling sensitivity of genotypes or according to the chilling treatment at 2 °C for 24 h.

**Figure 3:**
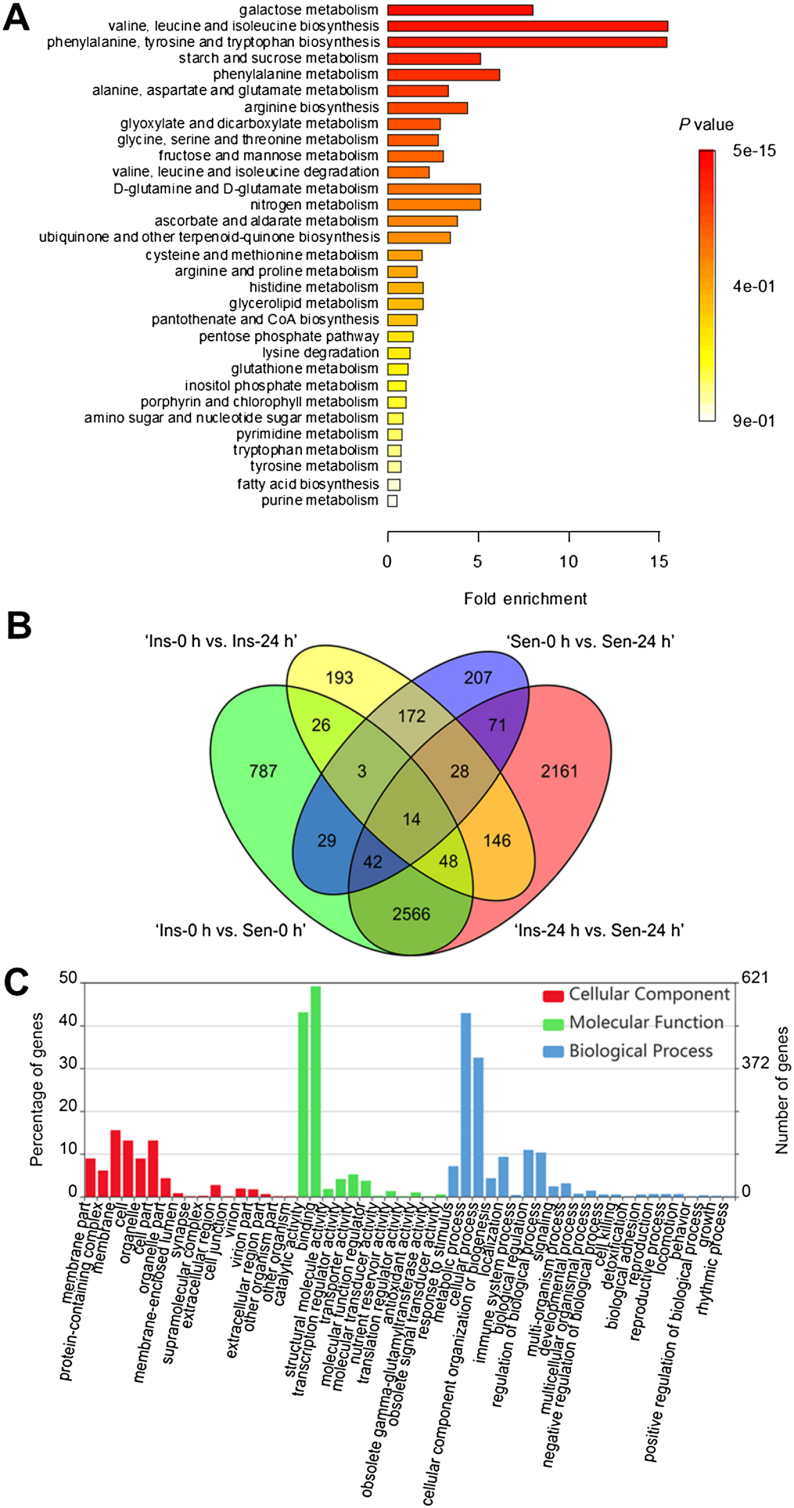
Metabolite set enrichment (MSE) and differentially expressed genes (DEGs) (**A**–**C**). MSE analysis (**A**), Venn diagrams of DEGs (**B**), and gene ontology annotation (**C**) of seeds obtained from ‘*UZB-GJG-1999-51*’ (chilling-insensitive, Ins) and ‘*C00562*’ (chilling-sensitive, Sen) peppers after chilling treatment at 2 °C for 0 h and 24 h, respectively. The numbers marked in the diagram indicate the number of significantly up- and downregulated genes among the four DEG sets (log twofold change > 1.0 and FDR-corrected *p* value < 0.05). (**A**) was created with freely available MetaboAnalyst (version 3.0, https://www.metaboanalyst.ca/).

To reveal the biological pathways involved in a chilling-induced seed browning in peppers by chilling treatment at 2 °C for 24 h, metabolite enrichment was analyzed (Fig. 3A). As a result, galactose metabolism; valine, leucine, and isoleucine biosynthesis; phenylalanine, tyrosine, and tryptophan biosynthesis; and starch and sucrose metabolism pathways in peppers were determined to be significantly affected by the chilling treatment at 2 °C for 24 h.

### Differentially expressed genes (DEGs)

Through the transcriptome analysis of seeds obtained from chilling-insensitive ‘*UZB-GJG-1999-51*’ and chilling-sensitive ‘*C00562*’ before or after chilling treatment, an average of 23.9 million reads ranging from 15.1 to 36.8 million reads among 12 samples (two genotypes + before and after chilling + 3 biological replicates) were obtained. On average, 82.7% of total reads were mapped to the transcript library. Furthermore, the percentages of Q30 base in all samples were higher than 96.50% and the percentages of Q20 were higher than 98.03% (Supplementary Table S3).

In total, 84,627 contigs were assembled with a reference genome (Pepper_Zunla_1_Ref_v1.0_rna.fna, accession number: GCF_000710875.1). The average contig length was 1010.6 bp, and the N50 was 1518 bp. To confirm the relationships among each sample and replicate, PCA and PLS-DA were performed using the changes of contigs (Supplementary Fig. S2). In the PCA score plot and PLS-DA score plot, the sum of first and second principal components accounted for 69.5% and 68.9% of the variance in the dataset, respectively. Also, the results of hierarchical analysis showed a clear distinction between the two pepper genotypes and the chilling treatment (Supplementary Fig. S3).

To minimize false positives, DEGs were selected using a threshold of ≥ twofold upregulated or downregulated genes with an FDR < 0.05. As a result, a total of 6493 contigs were defined as DEGs of chilling-insensitive (Ins) ‘*UZB-GJG-1999-51*’ and chilling-sensitive (Sen) ‘*C00562*’ (Fig. 3B). Gene ontology (GO) term and Kyoto Encyclopedia of Genes and Genomes (KEGG) enrichment were performed using 3,140 DEGs to identify the differences in specific gene expressions induced by the chilling treatment at 2 °C for 24 h. As an exception, 3353 contigs belong to only ‘Ins-0 h vs. Sen-0 h’ (787 contigs), and only in an intersection between ‘Ins-0 h vs. Sen-0 h’ and ‘Ins-24 h vs. Sen-24 h’ (2566 contigs).

Upregulated or downregulated biological functions by the application of chilling treatment at 2 °C for 24 h were investigated with GO-based enrichment analysis (Fig. 3C). The top 5, significantly enriched GO terms in the cellular component category were ‘intracellular (GO:0005622)’, ‘intracellular part (GO:0044424)’, ‘intracellular organelle (GO:0043229)’, ‘cell part (GO:0044464)’, and ‘membrane part (GO:0044425)’. Also, the top 5 enriched GO terms in the molecular function category were ‘protein binding (GO:0005515)’, ‘small molecule binding (GO:0036094)’, ‘ion binding (GO:0043167)’, ‘organic cyclic compound binding (GO:0097159)’, and ‘transferase activity (GO:0016740)’. Additionally, ‘primary metabolic process (GO:0044238)’, ‘nitrogen compound metabolic process (GO:0006807)’, ‘biosynthetic process (GO:0009058)’, ‘cellular metabolic process (GO:0044237)’, and ‘organic substance metabolic process (GO:0071704)’ belonged to the biological process category.

### Protein–protein interaction (PPI) network

DEGs were used to construct the PPI network which was composed of 760 nodes and 228 edges (Supplementary Fig. S4). Among them, 11 of the JA-related genes including LOX, AOC, JAR1, JAZ1, and JAZ3 were highlighted by a colored circle in Supplementary Fig. S4A. Also, except for PKT3, all of the JA-related genes were involved in abiotic, stress-related gene networks (Supplementary Fig. S4B). Therefore, it could be inferred that JA signaling is activated in the two genotypes; chilling-insensitive ‘*UZB-GJG-1999-51*’ and sensitive ‘*C00562*’.

### Heat-map of DEGs

**Figure 4:**
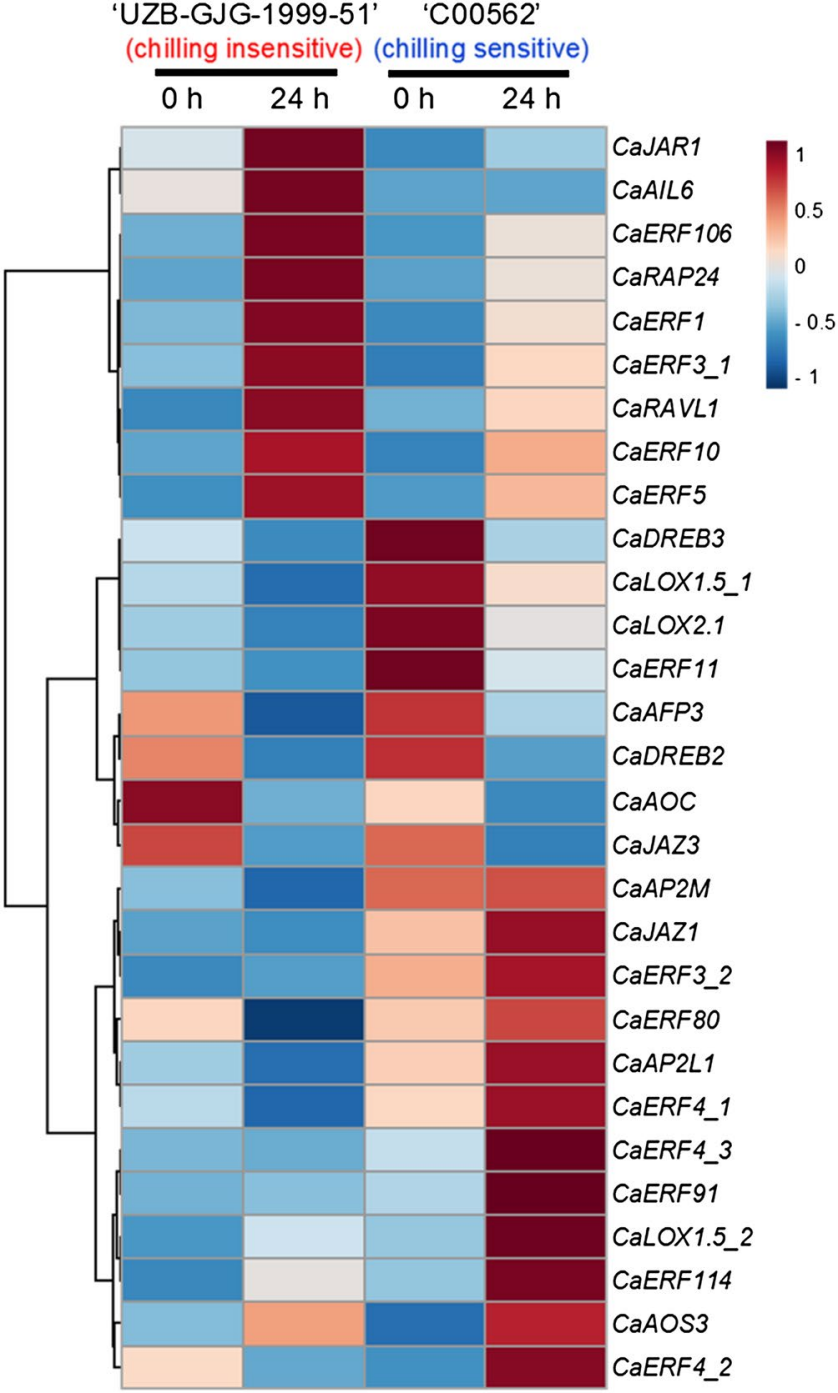
Heat-map of differentially expressed genes (DEGs). DEGs in jasmonic acid related genes and AP2/ERF family genes of seeds obtained from ‘*UZB-GJG-1999-51*’ (chilling-insensitive) and ‘*C00562*’ (chilling-sensitive) peppers after chilling treatment at 2 °C for 0 h and 24 h, respectively. This figure was created with freely available MetaboAnalyst (version 3.0, https://www.metaboanalyst.ca/).

To better understand the molecular responses of a chilling-induced seed browning of pepper fruit, a heat-map analysis was conducted using 29 DEGs consisting of 20 kinds of AP2/ERF family genes and 9 kinds of JA-related genes (Fig. 4 and Supplementary Table S4). Heat-map analysis clearly showed the differences in expression levels of 29 DEGs between chilling-insensitive ‘*UZB-GJG-1999-51*’ and chilling-sensitive ‘*C00562*’ before and after chilling treatment at 2 °C for 0 h and 24 h (Fig. 4). Supplementary Fig. S5 shows the correlation and correlation network analysis of 51 metabolites and 29 DEGs of seeds obtained from ‘*UZB-GJG-1999-51*’ and ‘*C00562*’ peppers.

Among the 20 kinds of AP2/ERF families, except for *CaDREB3* and *CaERF11*, the expression levels of *CaERF106*, *CaRAP24*, *CaERF1*, *CaERF3*_1, *CaRAVL1*, *CaERF10*, and *CaERF5* were upregulated by chilling treatment at 2 °C in the two genotypes (Fig. 4). Their expression levels were more highly upregulated in chilling-insensitive ‘*UZB-GJG-1999-51*’ than in chilling-sensitive ‘*C00562*’. However, the expression levels of the other AP2/ERF families of *CaERF3_2*, *CaERF80*, *CaAP2L1*, *CaERF4_1*, *CaERF4_3*, *CaERF91*, *CaERF114*, and *CaERF4_2* were upregulated in chilling-sensitive ‘*C00562*’ by the chilling treatment at 2 °C, but they were not upregulated in chilling-insensitive ‘*UZB-GJG-1999-51*’.

The expression level of *CaJAR1*, a JA-Ile biosynthesis gene, was up-regulated by chilling treatment in the two pepper genotypes, ‘*UZB-GJG-1999-51*’ and ‘*C00562*’ (Fig. 4). A higher *CaJAR1* expression level was observed in chilling-insensitive ‘*UZB-GJG-1999-51*’ than in chilling-sensitive ‘*C00562*’. Overall expression trends of *CaDREB3* and *CaERF11* were opposite to the overall expression trends of *CaJAR1*, *CaERF1*, *CaERF3_1*, *CaERF10*, and *CaERF5*. The levels of *CaDREB3* and *CaERF11* were more highly expressed in chilling-sensitive ‘*C00562*’ than in chilling-insensitive ‘*UZB-GJG-1999-51*’ and the expression levels of *CaDREB3* and *CaERF11* were downregulated by the chilling treatment at 2 °C in both genotypes.

### Relative gene expression levels

For quantitative PCR (qPCR), we analyzed seeds obtained from 6 additional pepper genotypes; 3 chilling-insensitive pepper fruits (‘*UZB-GJG-1999-51*’, ‘Takanotsume’, and ‘Hungarian Wax’), and 3 chilling-sensitive pepper fruits (‘*C00562*’, ‘Chili bangi’, and ‘Gyeonggiyangpyeong’) (Supplementary Fig. S1 and Supplementary Table S1).

**Figure 5:**
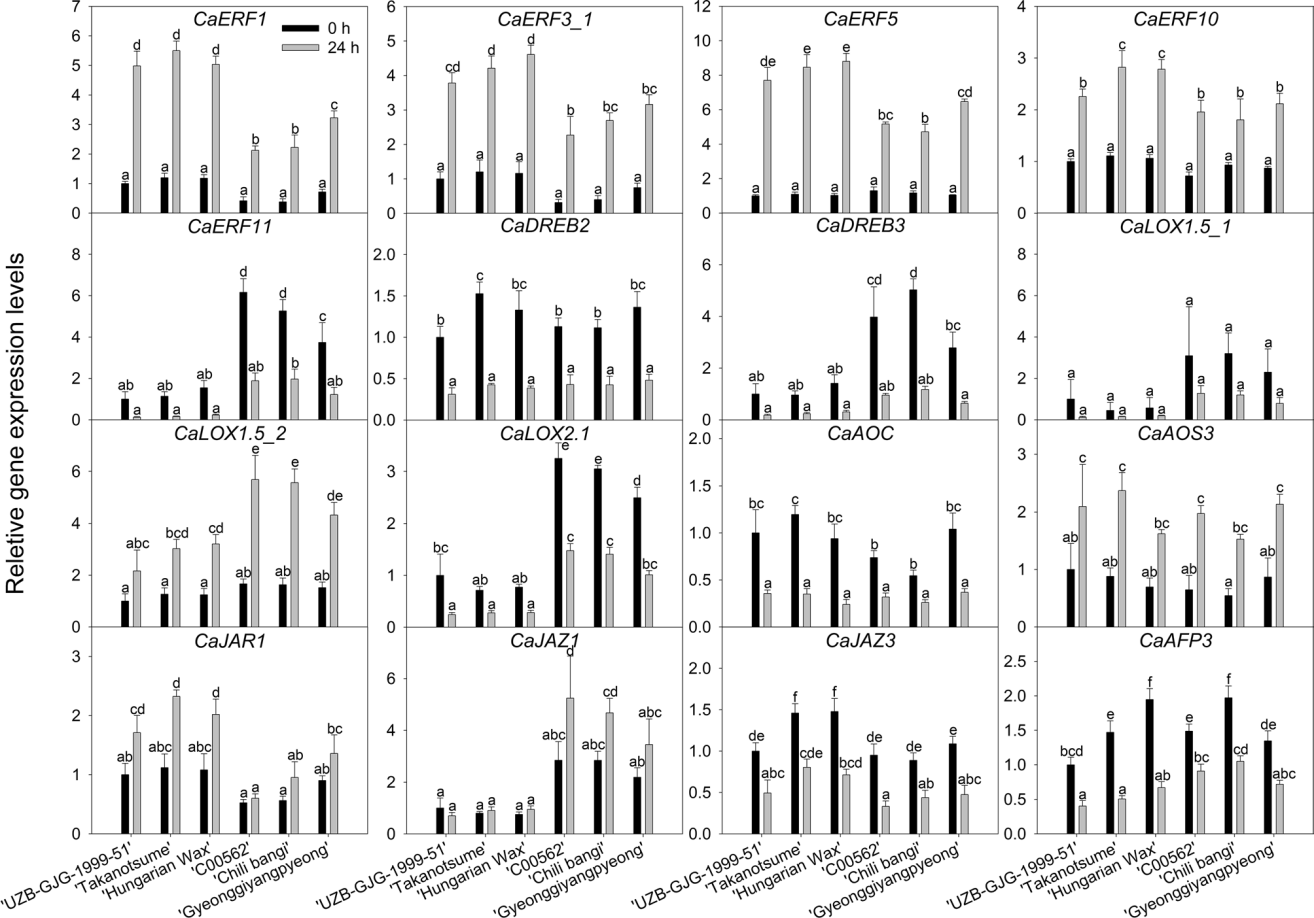
Relative expression levels of AP2/ERF family genes and jasmonic acid-related genes. Analysis of seeds obtained from chilling-insensitive ‘*UZB-GJG-1999-51*’, ‘Takanotsume’, ‘Hungarian Wax’ pepper fruit; and chilling-sensitive ‘*C00562*’, ‘Chili baggi’, and ‘Gyeonggiyangpyeong’ pepper fruit after chilling treatment at 2 °C for 0 h and 24 h, respectively. Vertical bars are means ± SD (n = 3). Different letters represent significant difference (*p* < 0.05) in Duncan’s multiple range test. The seed browning rate of each genotype is shown in Supplementary Table S1.

As shown in the results of the relative gene expression studies (Fig. 5), we could find clear differences between the chilling-insensitive and chilling-sensitive pepper fruits both before and after the chilling treatment. Analyzed genes were characterized into four categories: (1) genes that originally showed lower expression levels in insensitive genotypes were *CaEFR11, CaDREB3*, *CaLOX2.1*, and *CaJAZ1*; (2) genes that originally showed higher expression levels in insensitive genotypes were *CaERF1*, *CaEFR3_1*, *CaERF5*, *CaERF10*, and *CaJAR1*; (3) genes that showed significantly upregulated expression levels by chilling treatment were *CaERF1*, *CaERF3_1*, *CaERF5*, *CaERF10, CaLOX1.5_2*, *CaAOS3*, and *CaJAR1*; (4) conversely, genes that showed significantly downregulated expression levels by chilling treatment were *CaERF11*, *CaDREB2*, *CaDREB3*, *CaLOX2.1*, *CaAOC*, *CaJAZ3*, and *CaAFP3.*

The expression levels of transcription factors *CaERF1*, *CaERF3_1*, *CaERF5*, and *CaERF10* were significantly upregulated by chilling treatment in the 6 pepper genotypes (Fig. 5). At 0 h, before the chilling treatment, their expression levels were similar regardless of the genotypes. However, at 24 h, after chilling treatment, their expression levels were substantially higher in the chilling-insensitive genotypes of ‘*UZB-GJG-1999-51*’, ‘Takanotsume’, and ‘Hungarian Wax’ than in the chilling-sensitive genotypes of ‘*C00562*’, ‘chili bangi’, and ‘Gyeonggiyangpyeong’.

At 0 h, the relative expression levels of *CaERF11*, *CaDREB3*, and *CaLOX2.1* were significantly higher in the chilling-sensitive genotypes than in the chilling-insensitive genotypes (Fig. 5). However, their expression levels were substantially down-regulated in the 6 pepper genotypes after chilling treatment for 24 h. Chilling-insensitive genotypes showed substantially lower expression levels of *CaERF11*, *CaDREB3*, and *CaLOX2.1* than chilling-sensitive genotypes. Overall, their expression trends were very similar to each other, and the responses of their cellular mechanisms to chilling might be closely connected to each other in pepper fruit. We could assume that *CaERF11* and *CaDREB3* transcription factors act as negative regulators in the chilling response of pepper fruit.

*CaLOX1.5_1* expression levels were not different, however. The expression levels of *CaLOX1.5_2* were significantly upregulated in the chilling-insensitive and chilling-sensitive genotypes after chilling treatment (Fig. 5). The expression of *CaLOX1.5_2* was more highly upregulated in chilling-sensitive genotypes than in insensitive genotypes. The expression levels of *CaLOX1.5_2* showed similar levels in all peppers at 0 h.

The *CaJAR1* expressions were opposite to the *CaERF11* and *CaDREB3* expressions (Fig. 5). *CaJAR1* expressions were significantly upregulated in chilling-insensitive genotypes by the chilling treatment, and were slightly upregulated in chilling-sensitive genotypes. Chilling-insensitive groups originally had higher *CaJAR1* expressions than chilling-sensitive groups.

The levels of *CaDREB2*, *CaAOC*, *CaJAZ3*, and *CaAFP3* expressions were significantly higher in the 6 pepper genotypes at 0 h, before the chilling treatment (Fig. 5). However, they were clearly downregulated after the chilling treatment. Contrary to the expression levels of *CaDREB2*, *CaAOC*, *CaJAZ3*, and *CaAFP3*, the expression level of *CaAOS3* was significantly upregulated by the chilling treatment but there were no differences according to the genotypes. The chilling treatment upregulated the expression level of *CaJAZ1* only in chilling-sensitive groups, but not in chilling-insensitive groups.

**Figure 6:**
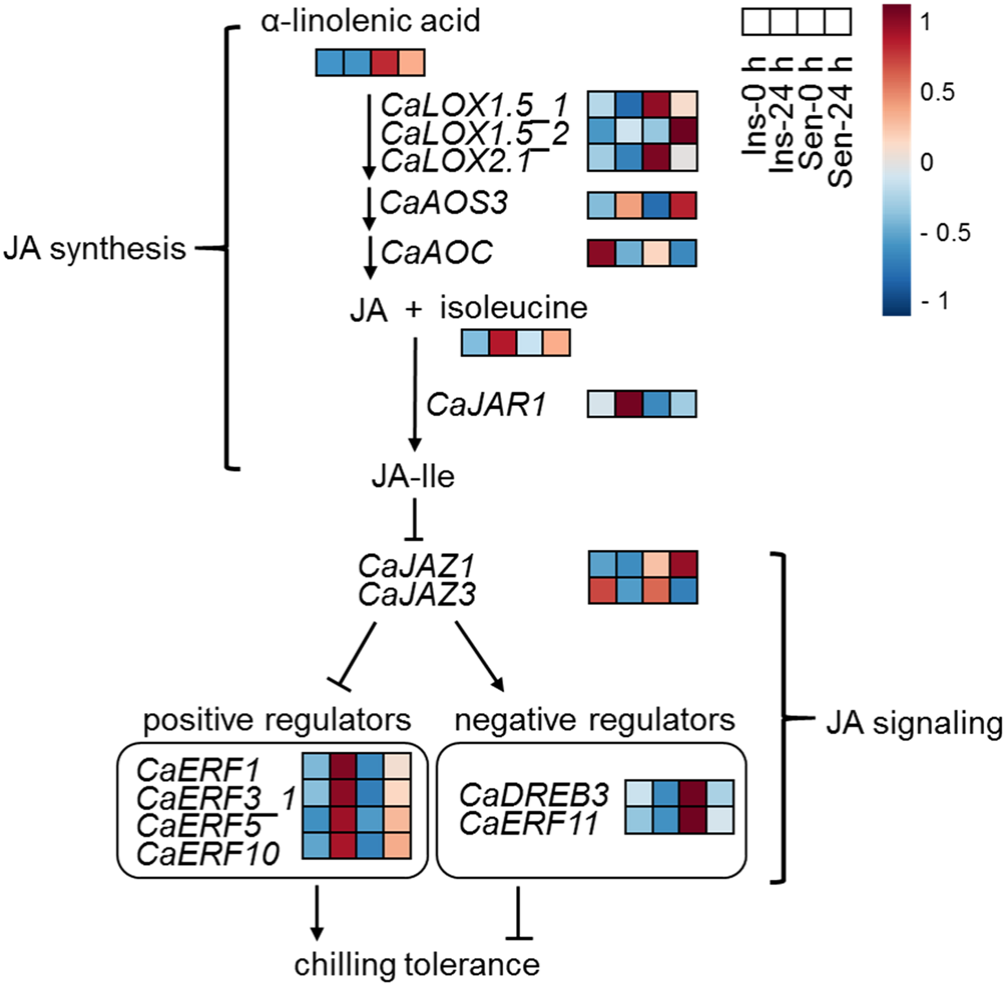
Putative chilling response pathway predicted in *Capsicum annuum* based on RNA-sequencing data. Chilling insensitive (Ins) ‘*UZB-GJG-1999-51*’ and chilling sensitive (Sen) ‘*C00562*’ peppers were treated with a chilling temperature of 2 °C for 0 h and 24 h after harvest, respectively.

Figure 6 shows the putative chilling response pathway predicted in *C. annuum* exposed to chilling conditions. Chilling-insensitive pepper fruit synthesizes more JA-Ile by higher upregulation of JAR1, and by higher rates of increasing isoleucine content than chilling-sensitive pepper fruit under chilling conditions. The transcription factors of *CaERF1*, *CaERF3_1*, *CaERF5*, and *CaERF10* might act as positive regulators to increase the chilling tolerance by showing higher expression levels in chilling-insensitive pepper fruit than in chilling-sensitive pepper fruit. However, *CaDREB3* and *CaERF11* transcription factors might act as negative regulators by showing highly upregulated expression levels in sensitive pepper fruit before chilling as well as by showing downregulated expression levels after chilling.

## Discussion

We analyzed a total of 51 targeted and untargeted metabolites of seeds obtained from chilling-insensitive ‘*UZB-GJG-1999-51*’ and chilling-sensitive ‘*C00562*’ peppers exposed to 2 °C for 24 h, and confirmed the changes of various metabolite levels (Fig. 1 and Fig. 2A). Our PLS-DA analysis indicated that pepper fruit are apparently separated according to their genotypes (PC 1); however, they are not clearly separated according to the chilling exposure time of 24 h (PC 2) (Fig. 1B). Metabolites are the end products of cell biology regulation processes and are closely related to phenotypes. Their levels can be regarded as the response of plant development to genetic and environmental changes^25,26^. Our results indicate that chilling-insensitive and sensitive pepper genotypes show differentially expressed metabolites, and that their levels are affected by chilling. We found that chilling exposure for 24 h was not enough to induce significant changes in all metabolites that were directly associated with the phenotype of seed browning induced by chilling.

The major differentially-expressed metabolites in the pepper fruit seeds were the organic acids; pyruvic acid, succinic acid, and fumaric acid. Their levels were significantly higher in chilling-sensitive ‘*C00562*’ peppers than in chilling-insensitive ‘*UZB-GJG-1999-51*’ peppers. Particularly, the pyruvic acid content was only significantly increased in chilling-sensitive ‘*C00562*’ peppers by the chilling treatment (Fig. 2A,B). According to a previous study, pyruvic acid was highly accumulated in cucumber and eggplant which were exposed to a chilling temperature of 1 °C^27^. Pyruvic acid increases the activity of alternative oxidase (AOX) within the cells^28^. Increased AOX gene expressions and AOX protein content then scavenges reactive oxygen species (ROS) generated under chilling conditions, resulting in higher chilling tolerance in the plant^4^. We speculate that the increased level of pyruvic acid in chilling-sensitive ‘*C00562*’ peppers after chilling treatment is probably due to the faster and more sensitive response to chilling stress compared to chilling-insensitive ‘*UZB-GJG-1999-51*’ peppers.

Increased pyruvic acid has been known to accelerate amino acid synthesis^29^. Among amino acids, BCAAs such as isoleucine, leucine, and valine are known to control abiotic stress responses induced by chilling^30^ through the regulation of osmotic pressure^31^. BCAA levels of leucine, isoleucine, and valine in rice seedlings increased to 6.8, 2.6, and 4.5 times higher than the initial level, respectively, after a chilling treatment at 4 °C for 24 h^32^. In this study, BCAA levels were significantly increased in seeds of pepper fruit treated with a chilling temperature of 2 °C at 24 h, regardless of the pepper genotype, and the increased levels were the same in both pepper genotypes. A difference in BCAA levels according to the chilling sensitivity of pepper fruit was not observed.

Similar to the result of the pyruvic acid analysis, palmitic acid, a major saturated fatty acid in pepper seeds, was significantly higher in chilling-sensitive ‘*C00562*’ than in chilling-insensitive ‘*UZB-GJG-1999-51*’, regardless of the chilling treatment (Fig. 2B). Also, it was only significantly increased in chilling-sensitive ‘*C00562*’ after the chilling treatment, but not in chilling-insensitive ‘*UZB-GJG-1999-51*’. Under chilling conditions, unsaturated fatty acids in cell membranes are usually converted to saturated fatty acids through the lipid peroxidation process by ROS^33^. In a comparison of pepper genotypes, chilling-insensitive ‘*UZB-GJG-1999-51*’ peppers are thought to be less prone to lipid peroxidation in chilling conditions because the palmitic acid level did not change even after the chilling treatment (Fig. 2B). On the other hand, chilling-sensitive ‘*C00562*’ has a rapid lipid peroxidation process as shown by a higher level of palmitic acid after chilling than chilling-insensitive ‘*UZB-GJG-1999-51*’. The difference in the degree of lipid peroxidation between the two genotypes might be a major factor in determining the chilling sensitivity of the two genotypes. In addition to the metabolite analysis, transcriptome analysis was performed to confirm a specific chilling response mechanism in pepper seeds.

Through transcriptome analysis of seeds obtained from chilling-insensitive and sensitive peppers treated at 2 °C for 24 h, a total of 84,627 contigs were identified. Our results of multivariate analysis using all contigs (Supplementary Fig. S2) and hierarchical analysis (Supplementary Fig. S3) show clear separations by pepper genotypes as well as by the chilling treatment. The effect of chilling treatment could be confirmed more reliably through transcriptome analysis than by metabolite analysis.

JA is involved in chilling responses by controlling ROS through *ERF* transcription factors^34^. However, the period of the chilling exposure for the activation of the JA synthesis pathway varies by plant. In *Arabidopsis*, JA content increased by 3 times after 1.5 h of chilling treatment at 4 °C and JA biosynthesis genes such as *LOX1_4*, *AOS*, and *AOC1_3* were significantly upregulated after 1.5 h of the chilling treatment^8^. Conversely, some crops require at least 24 h of chilling treatment to activate the JA synthesis pathway. For example, JA content in rice seedlings was increased by only 1.1 times after 1 day and increased by 2.0 times after 3 days of chilling treatment at 4 °C^35^. In addition, under a chilling treatment of 72 h or more, the expression levels of *LOX*, *AOS*, and *AOC* were significantly increased in *Camellia japonica* ‘Nuccio’s Bella Rossa’ compared to the other cultivars^36^.

In pepper fruit stored at 0 °C, the expression levels of *CaLOX*, *CaAOS*, and *CaAOC* were not different until 7 days of storage, but they were significantly different compared to the control after 14 days^37^. Similarly, in an experiment with *C. annuum* ‘Cheongyang’ stored at 2 °C for 25 days, *CaLOX* and *CaAOC* expression levels were not changed until 12 h, but the *CaAOC* expression level was significantly increased after 10 days during cold storage^5^. In this study, the expression levels of *CaLOX1.5_2* and *CaAOS3* were significantly increased after 24 h of the chilling treatment at 2 °C in both pepper genotypes (chilling-sensitive and insensitive peppers) (Fig. 5); however, the expression levels of *CaLOX2.1* and *CaAOC* were significantly decreased after chilling. These results might imply that the JA synthesis pathway in peppers was not perfectly activated in our experiment condition of chilling at 2 °C for 24 h.

The expression level of *CaJAR1* synthesizing JA-Ile, an active form of JA, was significantly increased in chilling-insensitive peppers but slightly increased in chilling-sensitive peppers (Fig. 5). Similarly, in *Arabidopsis*, the expression level of *JAR1* was increased by 8.1 times after chilling treatment at 4 °C for 24 h compared to before the treatment^8^. *JAR1* was more rapidly upregulated than *LOX1* and *AOC* in *Artemisia annua* treated with chilling at 4 °C^38^. In summary, we could assume that expression levels of *CaJAR1* in chilling-insensitive and sensitive peppers are very similar before chilling treatment but once pepper fruits are exposed to chilling, chilling-insensitive peppers increase the synthesis of JA-Ile, an active form of JA, by up-regulating *CaJAR1* in response to chilling stress.

Among the ERF family genes regulated by JA signaling, the *CaERF1*, *CaERF3_1*, *CaERF5*, and *CaERF10* expression levels were significantly increased by chilling treatment and found to be more significantly increased in chilling-insensitive genotypes. On the contrary, the expression levels of *CaERF11* and *CaDREB3* were significantly decreased in both genotypes by the chilling treatment (Fig. 5). *ERF1*, *ERF3*, and *ERF5* have been known to increase their expression levels under chilling stress in *Arabidopsis*^39^ and cotton^40^. In banana, *MaERF10* positively regulates chilling tolerance^41^. *DREB3* is also known to increase chilling tolerance in tomato^42,43^, but in this study we obtained the opposite result for *CaDREB3*. Therefore, further studies are needed to confirm whether the expression pattern of *CaDREB3* is specific to pepper fruit.

The involvement of *ERF11* in chilling responses has not been well reported. *ERF11* has been known to promote internode growth by activating gibberellin^44^ or to inhibit a stress response mechanism by antagonistically regulating *ERF6* in the plant^45^*. CaERF11* could be expected to play a role in suppressing the chilling tolerance of pepper fruit (Fig. 6).

In conclusion, the harvested pepper fruit in our study showed seed browning, which is a typical chilling symptom during cold storage or market distribution. Seed browning rates varied greatly according to pepper genotypes from chilling-insensitive (0.0%) to chilling-sensitive (over 60.0%). Integrating transcriptomic and metabolomics analysis clearly revealed differential metabolite accumulations and gene expressions between the chilling-insensitive and sensitive pepper genotypes. *CaERF1*, *CaERF3_1*, *CaERF5*, and *CaERF10* were found to be the key genes positively contributing to the chilling tolerance of insensitive pepper genotypes by showing significantly increased expressions under chilling conditions. However, *CaDREB3* and *CaERF11* always showed significantly higher expressions and were therefore found to be the key genes negatively contributing to the chilling tolerance of chilling-sensitive pepper genotypes. To the best of our knowledge, this study is the first to report on the molecular mechanisms regulating seed browning occurrence in *C. annuum* pepper fruit. These results can be used as a foundation for breeding pepper cultivars with high postharvest quality that show resistance to chilling stress. However, further research is required to confirm whether these *ERE* genes play a regulatory role in the chilling sensitivity of peppers, by up- or downregulating key genes.

## Materials and methods

### Plant materials and chilling treatment

We obtained seeds from 36 genotypes of *C. annuum* from the National Agrobiodiversity Center, Jeonju, Korea (Supplementary Table S1). Pepper seeds were planted in a greenhouse in Suwon, Korea. The plants were managed according to standard practices and fruits were harvested at maturity, approximately 45–50 days after full bloom, depending on the genotype. Pepper fruits of uniform size and color were harvested by hand for the experiment. They were immediately transported to the laboratory and then precooled at 18 °C for 8 h. After precooling, the fruits were transferred to storage chambers for cold storage at 2 °C for 3 weeks under 90% relative humidity and dark conditions.

Chilling injury symptoms in each genotype were carefully observed and photographed after cutting each fruit in half, lengthwise. This was done weekly during cold storage at 2 °C for 3 weeks. The seed browning rate of each fruit was calculated by the following equation and an average rate of seed browning for each genotype was determined. Twenty fruits were used as biological replicates in each pepper genotype.

$$ {\text{Seed }}\;{\text{browning }}\;{\text{rate }}\;{\text{of }}\;{\text{a }}\;{\text{pepper }}\left( \% \right) \, = \, \left[ {{\text{the }}\;{\text{number}}\;{\text{ of }}\;{\text{browned }}\;{\text{seeds}}/{\text{the}}\;{\text{ number }}\;{\text{of}}\;{\text{ total }}\left( {{\text{normal }} + {\text{ browned}}} \right){\text{ seeds}}} \right] \times {1}00 $$

For transcriptome and metabolomics studies, we selected two different pepper genotypes to find key genes involved in a chilling-induced seed browning based on our screening data of seed browning rates (Supplementary Table S1). The variety ‘*UZB-GJG-1999-51*’ was selected as a chilling-insensitive pepper (seed browning rate 0.00%) and ‘*C00562*’ as a chilling-sensitive pepper (seed browning rate 63.86%). Both ‘*UZB-GJG-1999-51*’ and ‘*C00562*’ fruits were exposed at 2 °C for 24 h without cold storage for 3 weeks. Pepper fruits were sampled before chilling treatment at 2 °C (0 h) and after chilling treatment for 24 h at 2 °C (24 h). All seeds obtained were collected without placenta, then immediately frozen in liquid nitrogen and kept at − 80 °C for all experiments.

For further verifying our results by qPCR analysis, two other chilling-insensitive peppers, ‘*Takanotsume*’ (seed browning rate 0.00%) and ‘*Hungarian Wax*’ (seed browning rate 4.31%); as well as two other chilling-sensitive peppers, ‘*Chili bangi*’ (seed browning rate 41.01%) and ‘*Gyeonggiyangpyeong*’ (seed browning rate 30.88%) (Supplementary Table S1) were also examined. They were sampled before the chilling treatment at 2 °C (0 h) and after the chilling treatment at 2 °C (24 h). All seeds obtained were also collected without placenta, then immediately frozen in liquid nitrogen and kept at − 80 °C for the qPCR analysis.

### Chemical reagents

All chemicals were obtained from Sigma-Aldrich (St. Louis, MO, USA) or Becton Dickinson (San Jose, CA, USA). All solvents used for HPLC were purchased from J.T. Baker (Center Valley, PA, USA).

### Free amino acids analysis

Free amino acids were analyzed following a previously described method^5,46^ with some modifications. First, frozen pepper seeds were completely ground into a fine powder using a mortar and pestle in liquid nitrogen. Then, 1.2 mL of 5% trichloroacetic acid was added to 100 mg of pepper seed powder and the mixture was sonicated at room temperature for 30 min. After reaching a concentration of 16,000×*g* at 4 °C, 1 mL of the supernatant was collected and filtered through a 0.45 μm polyvinylidene fluoride membrane filter. After mixing 5 μL of 0.4 N borate buffer (pH 10.2) and 1 μL of sample, 1 μL of *o*-phthalaldehyde, and 1 μL of fluorenylmethyloxycarbonyl were added for derivatization. Finally, 64 μL of distilled water was added and analyzed by HPLC. The column was equipped with a Zorbax eclipse AAA (4.6 × 150 mm, Agilent, Santa Clara, CA, USA) and the flow rate was set to 2 mL min^−1^. The mobile phase A was set to 40 mM NaH_2_PO_4_ (pH 7.8), and B was set to acetonitrile:methanol:H_2_O (45:45:10, v:v:v).

### Fatty acid analysis

Fatty acids were analyzed following a previously described method^47^ with some modifications. First, 100 mg of ground frozen pepper seed was placed in a Teflon cap tube and extracted with 2 mL of methylation mixture (methanol:benzene:2,2′-dimethoxypropane:H_2_SO_4_ = 39:20:5:2, v:v:v:v) and 1 mL of heptane at 80 °C for 2 h. After cooling at room temperature, the supernatant was taken and analyzed by GC. Agilent 7890A (Agilent, Santa Clara, CA, USA) was used and the column was DB-23 23 (0.25 mm × 60 m × 0.25 μm, Agilent, Santa Clara, CA, USA). The FID detector was set at 280 °C and the flow rates were 35 mL min^−1^ for H_2_, 350 mL min^−1^ for air, and 35 mL min^−1^ for He. The injector temperature was set at 250 °C. The oven temperature increased from 50 °C to 130 °C by 15 °C min^−1^, to 170 °C by 8 °C min^−1^, and to 215 °C by 2 °C min^−1^.

### Untargeted polar phase metabolite analysis

Polar phase compounds were extracted following a previously described method^5,48,49^ with some modifications. Fifty-mg of ground frozen pepper seeds was mixed with 1.2 mL of methanol and shaken at 75 °C for 30 min; then centrifuged at 13,500×*g* for 10 min. Next, 0.7 mL of the supernatant was transferred to a 2 mL-microtube and mixed with 0.5 mL of chloroform and 20 μL of ribitol (internal standard). Immediately after adding 0.7 mL of distilled water, it was centrifuged at 2500×*g* for 10 min. Then 0.5 mL of the supernatant was concentrated using a nitrogen evaporator (MG-2200, Eyela, Japan). Methoxyamine hydrochloride (50 μL) was added and incubated at 37 °C for 2 h. After incubation, 40 μL of sample and 100 μL of *N*-methyl-*N*-trifluoroacetamide were mixed and incubated at 37 °C for 30 min. Then, 1 µL of the sample was injected into the GC–MS ISQ LT system (Thermo Fisher Scientific, Waltham, MA, USA) using an auto sampler. The DB-5-fused silica capillary column (0.25 mm × 30 m × 0.25 μm, Agilent, Santa Clara, CA, USA) was used and the oven temperature was set to increase from 50 °C to 310 °C at a rate of 5 °C min^−1^. The injector was in the split-less mode at 250 °C. Helium was used as the carrier gas at a flow rate of 1 × 10^–3^ L min^−1^. The range of mass scan was from 35 to 550 m/z.

### Total RNA isolation, cDNA library construction, and sequencing

Frozen seeds were ground into a fine powder using a mortar and pestle in liquid nitrogen and 100 mg of powder was used for total RNA extraction. Total RNA was extracted using Ribospin Seed/Fruit Kit (GeneAll, Seoul, Korea) following the manufacturer’s instructions. Extracted total RNA was used for RNA-seq and cDNA synthesis. cDNA was synthesized using an amfiRivert Platinum cDNA Synthesis Master Mix Kit (GenDEPOT, Baker, TX, USA) following the manufacturer’s instructions.

### Transcriptome analysis by RNA-Seq

Total RNA from ‘*UZB-GJG-1999-51*’ and ‘*C00562*’ peppers treated with chilling for 0 h and 24 h were used for RNA-seq. RNA-seq was performed using the Illumina Hi-Seq 2500 system (Illumina, San Diego, CA, USA) with 151-bp paired-end at the National Instrumentation Center for Environmental Management (NICEM), Seoul National University, South Korea. As a pretreatment process, artifacts such as adaptor sequence, contaminant DNA, and PCR duplicates, were removed from raw reads. Then, preprocessed reads were assembled and mapped to Pepper Zunla 1 Ref_v1.0 reference genome (GCF_000710875.1, NCBI).

### Identification of DEGs

To identify DEGs between the two different genotypes, ‘*UZB-GJG-1999-51*’ and ‘*C00562*’ pepper fruits were treated with chilling for 0 h or 24 h, at 2 °C. The expression levels of each read were calculated according to the fragment per kilobase of transcript per million mapped reads (FPKM) value. DEGs were selected using a threshold of ≥ twofold upregulated or downregulated genes with an FDR < 0.05 and all processes were performed by EdgeR software.

### GO term and KEGG enrichment analyses for DEGs

Functional-enrichment analysis including GO and KEGG were performed using Blast2GO 5.2^50^ and MapMan 3.6.0 (https://mapman.gabipd.org), respectively.

### qPCR analysis

For qPCR, the cDNA was diluted tenfold. The qPCR was performed using 2X Real-Time PCR Master Mix (Biofact, Daejeon, Korea) in a final volume of 20 μL and CFX Connect Real-Time System (BIO-RAD, Hercules, CA, USA) under the following conditions: 95 °C for 15 min followed by 40 cycles of 95 °C for 20 s, 55 °C for 40 s, and 72 °C for 20 s. Relative gene expressions were determined with normalization against the expression of the pepper *CaActin7* gene. The primers used for the qPCR are listed in Supplementary Table S2, and are designed based on a reference gene set using the Primer 3 plus server (https://www.bioinformatics.nl/cgi-bin/primer3plus/primer3plus.cgi). The relative gene expression was calculated using the 2^-ΔΔCt^ method^51^.

### Statistical analysis

All experimental analyses were conducted in randomized designs with three biological replicates (n = 3). Statistical comparison between the means of the experimental groups was carried out using SPSS ver. 22.0 (IBM, Armonk, NY, USA). One-way analysis of variance and Duncan’s multiple range test were performed to determine significant differences. The metabolite data were auto-scaled and used for PCA, PLS-DA, heat-map analysis, and metabolite enrichment analysis with MetaboAnalyst 3.0 software (www.metaboanalyst.ca), and PPI analysis and correlation network analysis with Cytoscape v3.6.1 software (https://cytoscape.github.io/).

## Supplementary information

Supplementary Figures.

Supplementary Tables.

## Data Availability

All datasets generated for this study are included in the article and Supplementary Materials.

## References

[1] Özden Ç, Bayindirli L (2002). Effects of combinational use of controlled atmosphere, cold storage and edible coating applications on shelf life and quality attributes of green peppers. Eur. Food Res. Technol..

[2] Valenzuela JL (2017). Oxidative stress associated with chilling injury in immature fruit: postharvest technological and biotechnological solutions. Int. J. Mol. Sci..

[3] Boonsiri K, Ketsa S, van Doorn WG (2007). Seed browning of hot peppers during low temperature storage. Postharvest Biol. Technol..

[4] Fung RWM, Wang CY, Smith DL, Gross KC, Tian M (2004). MeSA and MeJA increase steady-state transcript levels of alternative oxidase and resistance against chilling injury in sweet peppers (*Capsicum annuum* L.). Plant Sci..

[5] Seo J, Yi G, Lee JG, Choi JH, Lee EJ (2020). Seed browning in pepper (*Capsicum annuum* L.) fruit during cold storage is inhibited by methyl jasmonate or induced by methyl salicylate. Postharvest Biol. Technol..

[6] Fonseca S (2009). (+)-7-iso-Jasmonoyl-L-isoleucine is the endogenous bioactive jasmonate. Nat. Chem. Biol..

[7] Hu Y (2017). Jasmonate regulates leaf senescence and tolerance to cold stress: crosstalk with other phytohormones. J. Exp. Bot..

[8] Hu Y, Jiang L, Wang F, Yu D (2013). Jasmonate regulates the inducer of CBF expression–c-repeat binding factor/DRE binding factor1 cascade and freezing tolerance in *Arabidopsis*. Plant Cell..

[9] Wang F (2016). Phytochrome A and B function antagonistically to regulate cold tolerance via abscisic acid-dependent jasmonate signaling. Plant Cell Physiol..

[10] Pratiwi P (2017). Identification of jasmonic acid and jasmonoyl-isoleucine, and characterization of AOS, AOC, OPR and JAR1 in the model lycophyte Selaginella moellendorffii. Plant Cell Physiol..

[11] Zhang F (2015). Structural basis of JAZ repression of MYC transcription factors in jasmonate signalling. Nature.

[12] Pieterse CM, Pierik R, Van Wees SC (2014). Different shades of JAZ during plant growth and defense. New Phytol..

[13] Pauwels L, Goossens A (2011). The JAZ proteins: a crucial interface in the jasmonate signaling cascade. Plant Cell..

[14] Melotto M (2008). A critical role of two positively charged amino acids in the Jas motif of *Arabidopsis* JAZ proteins in mediating coronatine-and jasmonoyl isoleucine-dependent interactions with the COI1 F-box protein. Plant J..

[15] Lorenzo O, Piqueras R, Sánchez-Serrano JJ, Solano R (2003). ETHYLENE RESPONSE FACTOR1 integrates signals from ethylene and jasmonate pathways in plant defense. Plant Cell..

[16] Sears MT (2014). NtERF32: a non-NIC2 locus AP2/ERF transcription factor required in jasmonate-inducible nicotine biosynthesis in tobacco. Plant Mol. Biol..

[17] Pré M (2008). The AP2/ERF domain transcription factor ORA59 integrates jasmonic acid and ethylene signals in plant defense. Plant Physiol..

[18] Xie Z, Nolan TM, Jiang H, Yin Y (2019). AP2/ERF transcription factor regulatory networks in hormone and abiotic stress responses in *Arabidopsis*. Front. Plant Sci..

[19] Bolt S, Zuther E, Zintl S, Hincha DK, Schmülling T (2017). *ERF105* is a transcription factor gene of *Arabidopsis thaliana* required for freezing tolerance and cold acclimation. Plant Cell Environ..

[20] Wang M (2019). ERF109 of trifoliate orange (*Poncirus trifoliata* (L.) Raf.) contributes to cold tolerance by directly regulating expression of Prx1 involved in antioxidative process. Plant Biotechnol. J..

[21] Sun X (2019). The ethylene response factor VaERF 092 from Amur grape regulates the transcription factor VaWRKY 33, improving cold tolerance. Plant J..

[22] Lv K (2020). Overexpression of an *AP2/ERF* family gene, *BpERF13*, in birch enhances cold tolerance through upregulating *CBF* genes and mitigating reactive oxygen species. Plant Sci..

[23] Leone M, Keller MM, Cerrudo I, Ballaré CL (2014). To grow or defend? Low red: far-red ratios reduce jasmonate sensitivity in *Arabidopsis* seedlings by promoting DELLA degradation and increasing JAZ10 stability. New Phytol..

[24] Lissarre M, Ohta M, Sato A, Miura K (2010). Cold-responsive gene regulation during cold acclimation in plants. Plant Signal. Behav..

[25] Fiehn O (2000). Metabolite profiling for plant functional genomics. Nat. Biotechnol..

[26] Lin W, Li Y, Lu Q, Lu H, Li J (2020). Combined analysis of the metabolome and transcriptome identified candidate genes involved in phenolic acid biosynthesis in the leaves of *Cyclocarya paliurus*. Int. J. Mol. Sci..

[27] Tsuchida H, Dan-Hong C, Inoue K, Kozukue N, Mizuno S (1990). Changes in pyruvic acid content and GPT activity in chilling-sensitive and nonsensitive crops. HortSci..

[28] Kumar N, Vyas D, Kumar S (2007). Plants at high altitude exhibit higher component of alternative respiration. J. Plant Physiol..

[29] Vanlerberghe GC, McIntosh L (1997). Alternative oxidase: from gene to function. Annu. Rev. Plant Physiol. Plant Mol. Biol..

[30] Obata T, Fernie AR (2012). The use of metabolomics to dissect plant responses to abiotic stresses. Cell. Mol. Life Sci..

[31] Joshi V, Joung JG, Fei Z, Jander G (2010). Interdependence of threonine, methionine and isoleucine metabolism in plants: accumulation and transcriptional regulation under abiotic stress. Amino Acids.

[32] Zhao XQ (2013). Temporal profiling of primary metabolites under chilling stress and its association with seedling chilling tolerance of rice (*Oryza sativa* L.). Rice..

[33] Liavonchanka A, Feussner I (2006). Lipoxygenases: occurrence, functions and catalysis. J. Plant Physiol..

[34] Sharma M, Laxmi A (2015). Jasmonates: emerging players in controlling temperature stress tolerance. Front. Plant Sci..

[35] Du H, Liu H, Xiong L (2013). Endogenous auxin and jasmonic acid levels are differentially modulated by abiotic stresses in rice. Front. Plant Sci..

[36] Li Q (2016). RNA-seq based transcriptomic analysis uncovers α-linolenic acid and jasmonic acid biosynthesis pathways respond to cold acclimation in *Camellia japonica*. Sci. Rep..

[37] Shin SY, Park MH, Choi JW, Kim JG (2017). Gene network underlying the response of harvested pepper to chilling stress. J. Plant Physiol..

[38] Liu W (2017). Cold stress improves the production of artemisinin depending on the increase in endogenous jasmonate. Biotechnol. Appl. Biochem..

[39] Qiao ZX, Huang B, Liu JY (2008). Molecular cloning and functional analysis of an ERF gene from cotton (*Gossypium hirsutum*). BBA Gene Regul. Mech..

[40] Jin LG, Li H, Liu JY (2010). Molecular characterization of three ethylene responsive element binding factor genes from cotton. J. Integr. Plant Biol..

[41] Qi XN (2016). A banana fruit transcriptional repressor MaERF10 interacts with MaJAZ3 to strengthen the repression of JA biosynthetic genes involved in MeJA-mediated cold tolerance. Postharvest Biol. Technol..

[42] Islam MS, Wang MH (2009). Expression of dehydration responsive element-binding protein-3 (DREB3) under different abiotic stresses in tomato. BMB Rep..

[43] Wang G (2019). A tomato transcription factor, SlDREB3 enhances the tolerance to chilling in transgenic tomato. Plant Physiol. Biochem..

[44] Zhou X (2016). The ERF11 transcription factor promotes internode elongation by activating gibberellin biosynthesis and signaling. Plant Physiol..

[45] Dubois M (2015). The ETHYLENE RESPONSE FACTORs ERF6 and ERF11 antagonistically regulate mannitol-induced growth inhibition in *Arabidopsis*. Plant Physiol..

[46] Park JH, Park JS, Choi JS (2014). Basic amino acid-conjugated polyamidoamine dendrimers with enhanced gene transfection efficiency. Macromol. Res..

[47] Garcés R, Mancha M (1993). One-step lipid extraction and fatty acid methyl esters preparation from fresh plant tissues. Anal. Biochem..

[48] Lim S, Lee JG, Lee EJ (2017). Comparison of fruit quality and GC–MS-based metabolite profiling of kiwifruit ‘Jecy green’: natural and exogenous ethylene-induced ripening. Food Chem..

[49] Bang J, Lim S, Yi G, Lee JG, Lee EJ (2019). Integrated transcriptomic-metabolomic analysis reveals cellular responses of harvested strawberry fruit subjected to short-term exposure to high levels of carbon dioxide. Postharvest Biol. Technol..

[50] Conesa A (2005). Blast2GO: a universal tool for annotation, visualization and analysis in functional genomics research. Bioinformatics.

[51] Livak KJ, Schmittgen TD (2001). Analysis of relative gene expression data using real-time quantitative PCR and the 2(-Delta Delta C(T)) method. Methods.

