# Supplementary material for: Jasmonic acid and ERF family genes are involved in chilling sensitivity and seed browning of pepper fruit after harvest
Source: Sci Rep. 2020 Oct 21;10:17949. doi: 10.1038/s41598-020-75055-z (PMC7577993; doi:10.1038/s41598-020-75055-z)
Supplement: Supplementary file 1 — Supplementary Figures. [file 41598_2020_75055_MOESM1_ESM.pptx]

## Slide 1
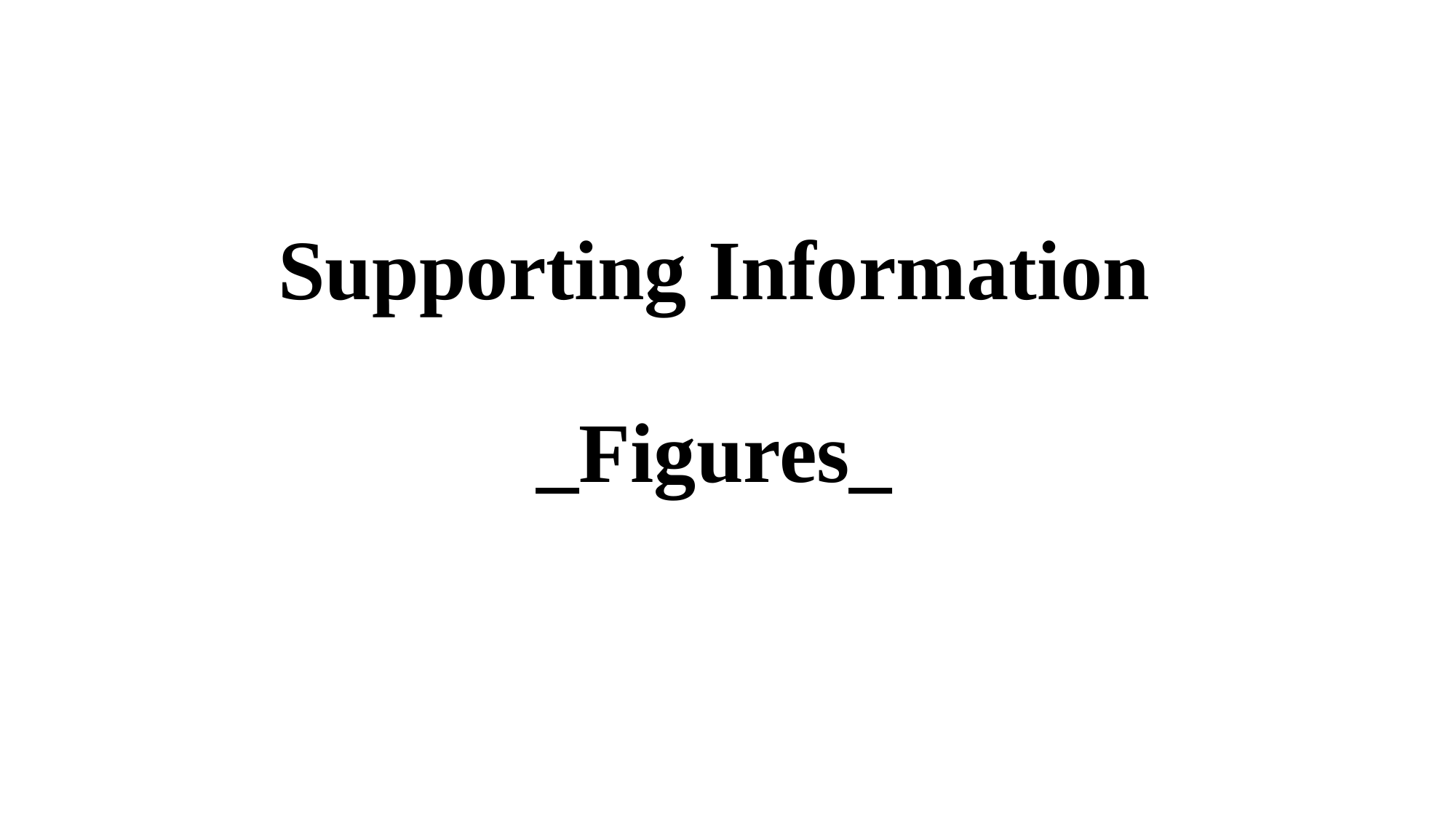

# Supporting Information _Figures_

## Slide 2
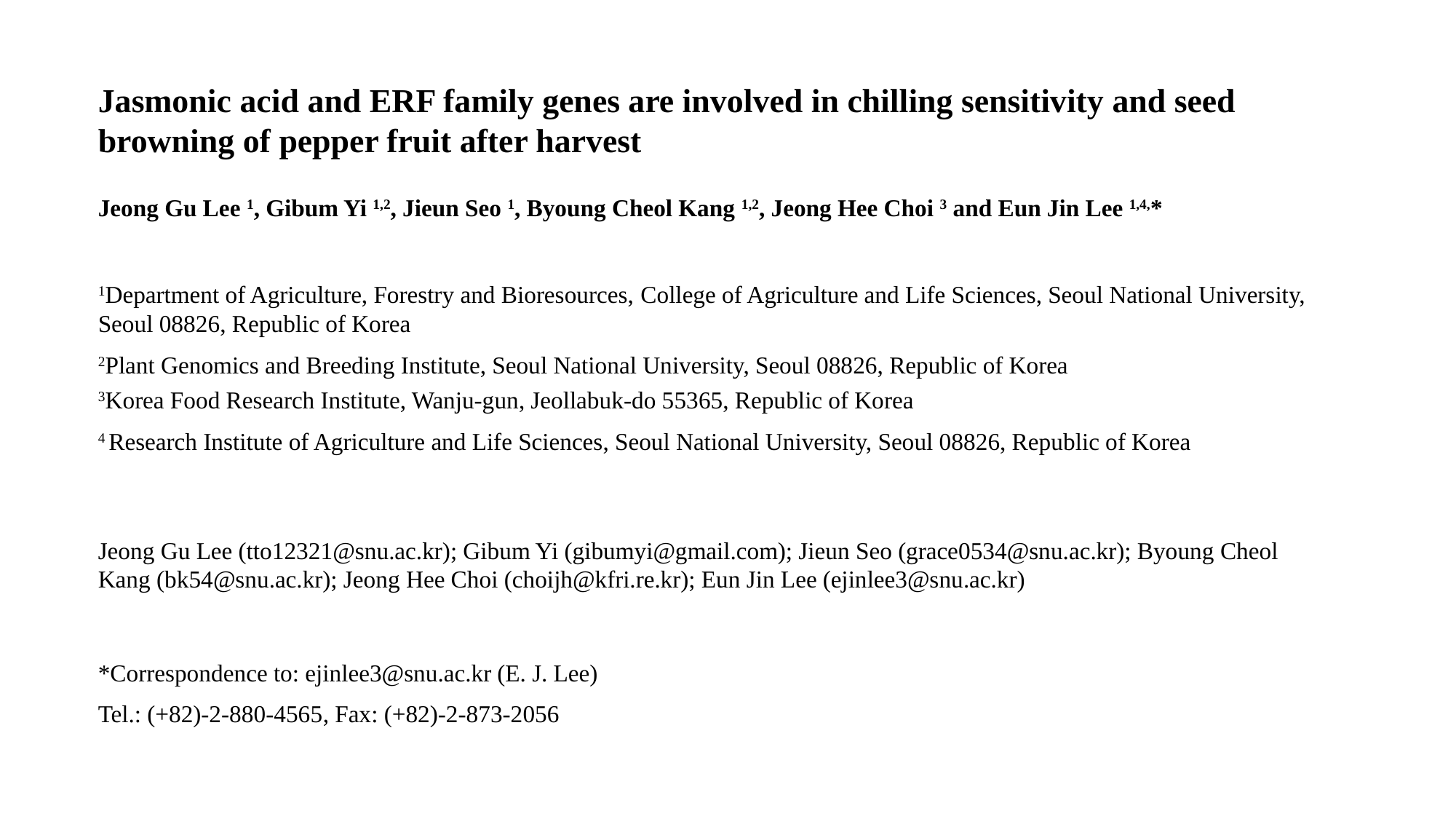

Jasmonic acid and ERF family genes are involved in chilling sensitivity and seed browning of pepper fruit after harvest
Jeong Gu Lee 1, Gibum Yi 1,2, Jieun Seo 1, Byoung Cheol Kang 1,2, Jeong Hee Choi 3 and Eun Jin Lee 1,4,*
1Department of Agriculture, Forestry and Bioresources, College of Agriculture and Life Sciences, Seoul National University, Seoul 08826, Republic of Korea
2Plant Genomics and Breeding Institute, Seoul National University, Seoul 08826, Republic of Korea
3Korea Food Research Institute, Wanju-gun, Jeollabuk-do 55365, Republic of Korea
4 Research Institute of Agriculture and Life Sciences, Seoul National University, Seoul 08826, Republic of Korea
Jeong Gu Lee (tto12321@snu.ac.kr); Gibum Yi (gibumyi@gmail.com); Jieun Seo (grace0534@snu.ac.kr); Byoung Cheol Kang (bk54@snu.ac.kr); Jeong Hee Choi (choijh@kfri.re.kr); Eun Jin Lee (ejinlee3@snu.ac.kr)
*Correspondence to: ejinlee3@snu.ac.kr (E. J. Lee)
Tel.: (+82)-2-880-4565, Fax: (+82)-2-873-2056

## Slide 3
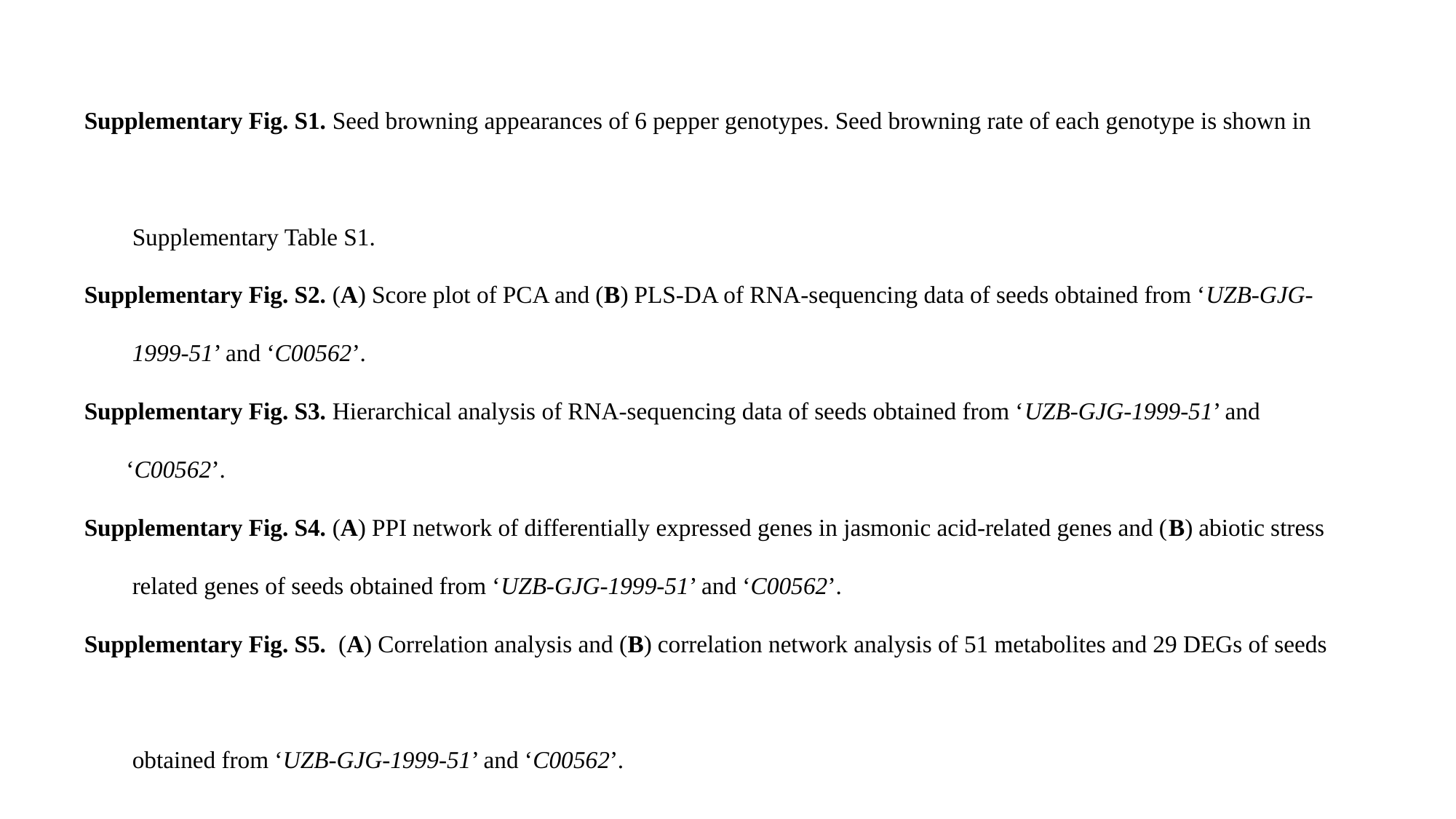

Supplementary Fig. S1. Seed browning appearances of 6 pepper genotypes. Seed browning rate of each genotype is shown in
 Supplementary Table S1.
Supplementary Fig. S2. (A) Score plot of PCA and (B) PLS-DA of RNA-sequencing data of seeds obtained from ‘UZB-GJG-
 1999-51’ and ‘C00562’.
Supplementary Fig. S3. Hierarchical analysis of RNA-sequencing data of seeds obtained from ‘UZB-GJG-1999-51’ and
 ‘C00562’.
Supplementary Fig. S4. (A) PPI network of differentially expressed genes in jasmonic acid-related genes and (B) abiotic stress
 related genes of seeds obtained from ‘UZB-GJG-1999-51’ and ‘C00562’.
Supplementary Fig. S5. (A) Correlation analysis and (B) correlation network analysis of 51 metabolites and 29 DEGs of seeds
 obtained from ‘UZB-GJG-1999-51’ and ‘C00562’.

## Slide 4
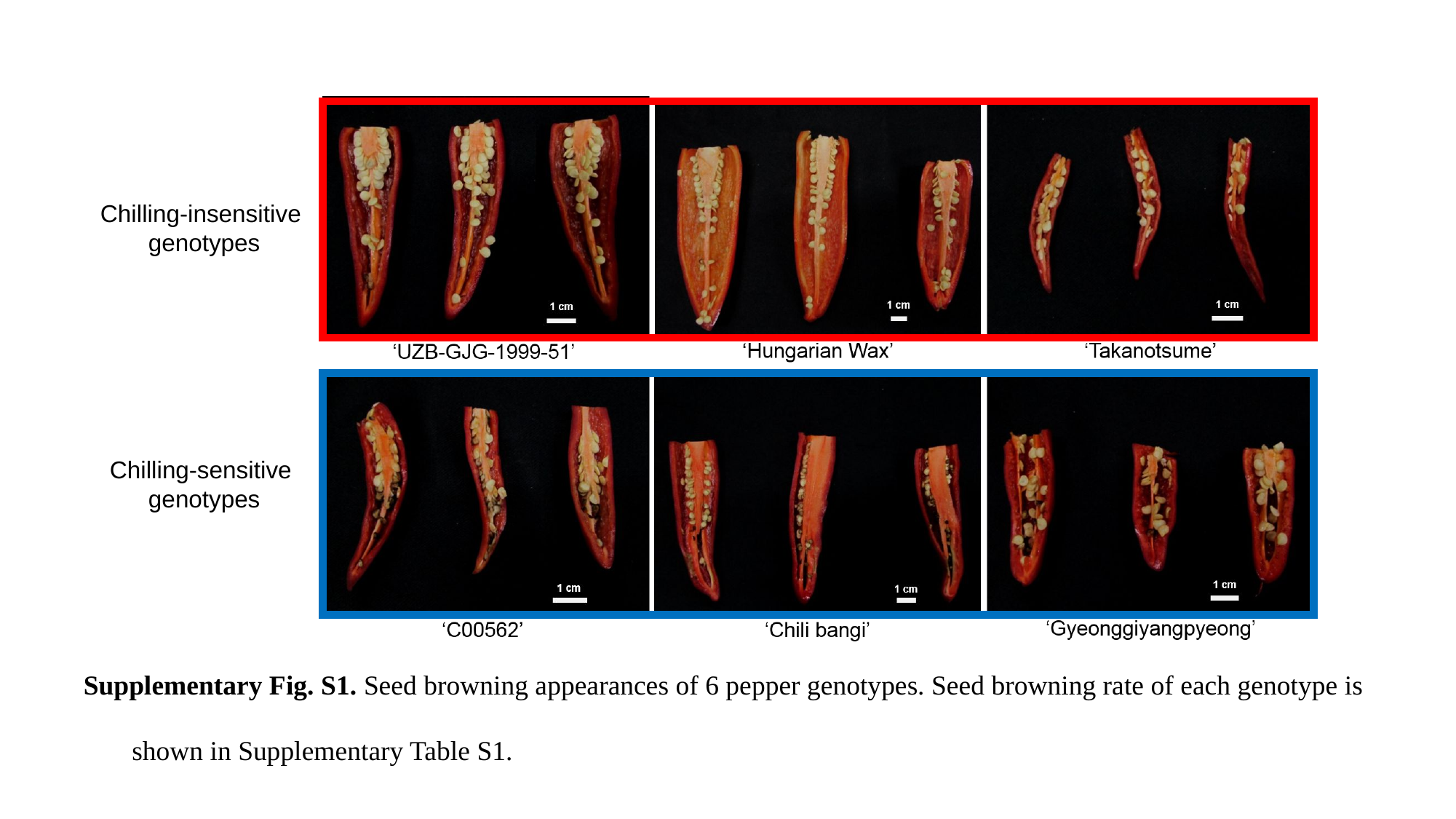

Chilling-insensitive
genotypes
Chilling-sensitive
genotypes
Supplementary Fig. S1. Seed browning appearances of 6 pepper genotypes. Seed browning rate of each genotype is
 shown in Supplementary Table S1.

## Slide 5
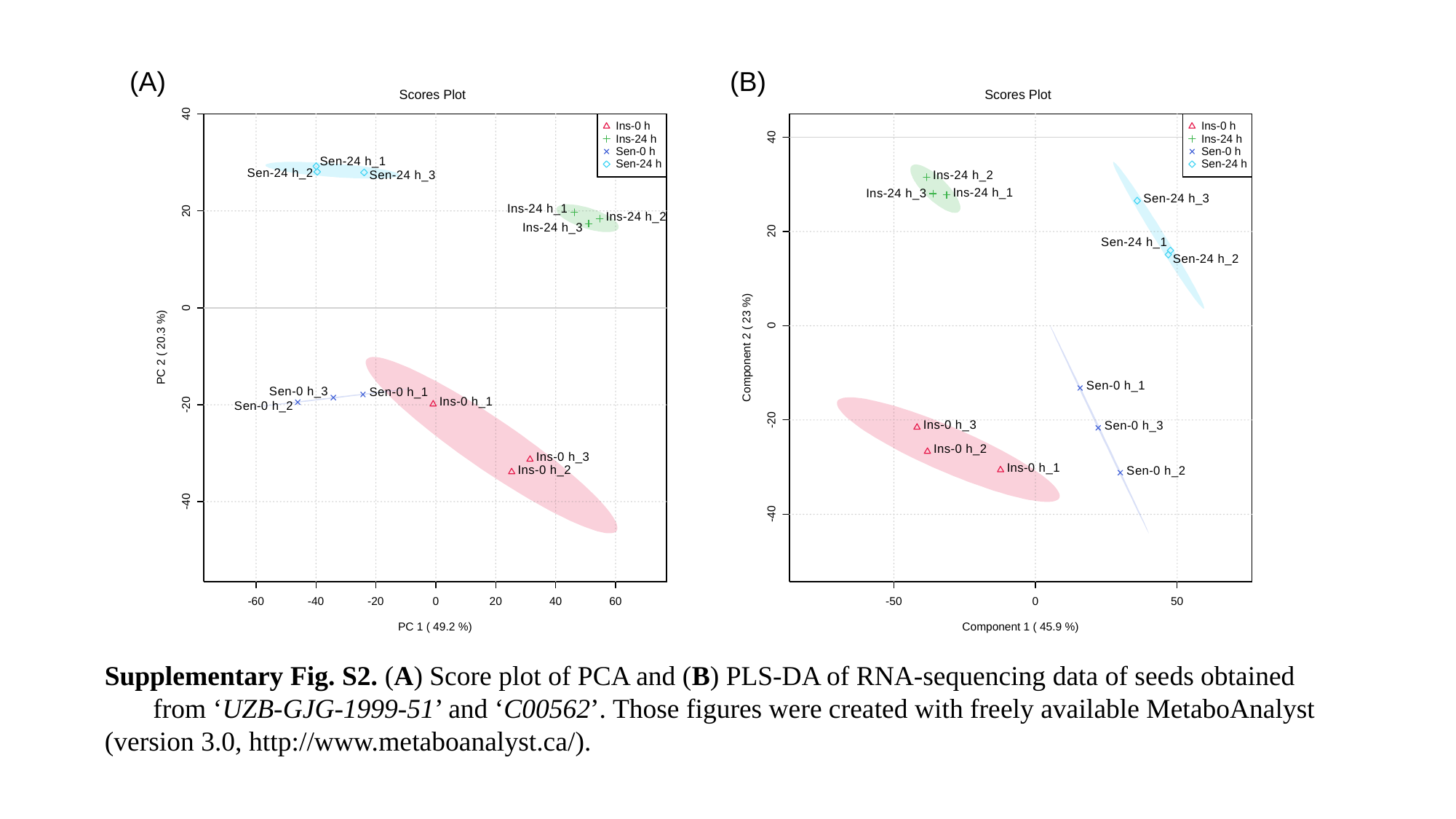

(A)
(B)
Supplementary Fig. S2. (A) Score plot of PCA and (B) PLS-DA of RNA-sequencing data of seeds obtained
 from ‘UZB-GJG-1999-51’ and ‘C00562’. Those figures were created with freely available MetaboAnalyst (version 3.0, http://www.metaboanalyst.ca/).

## Slide 6
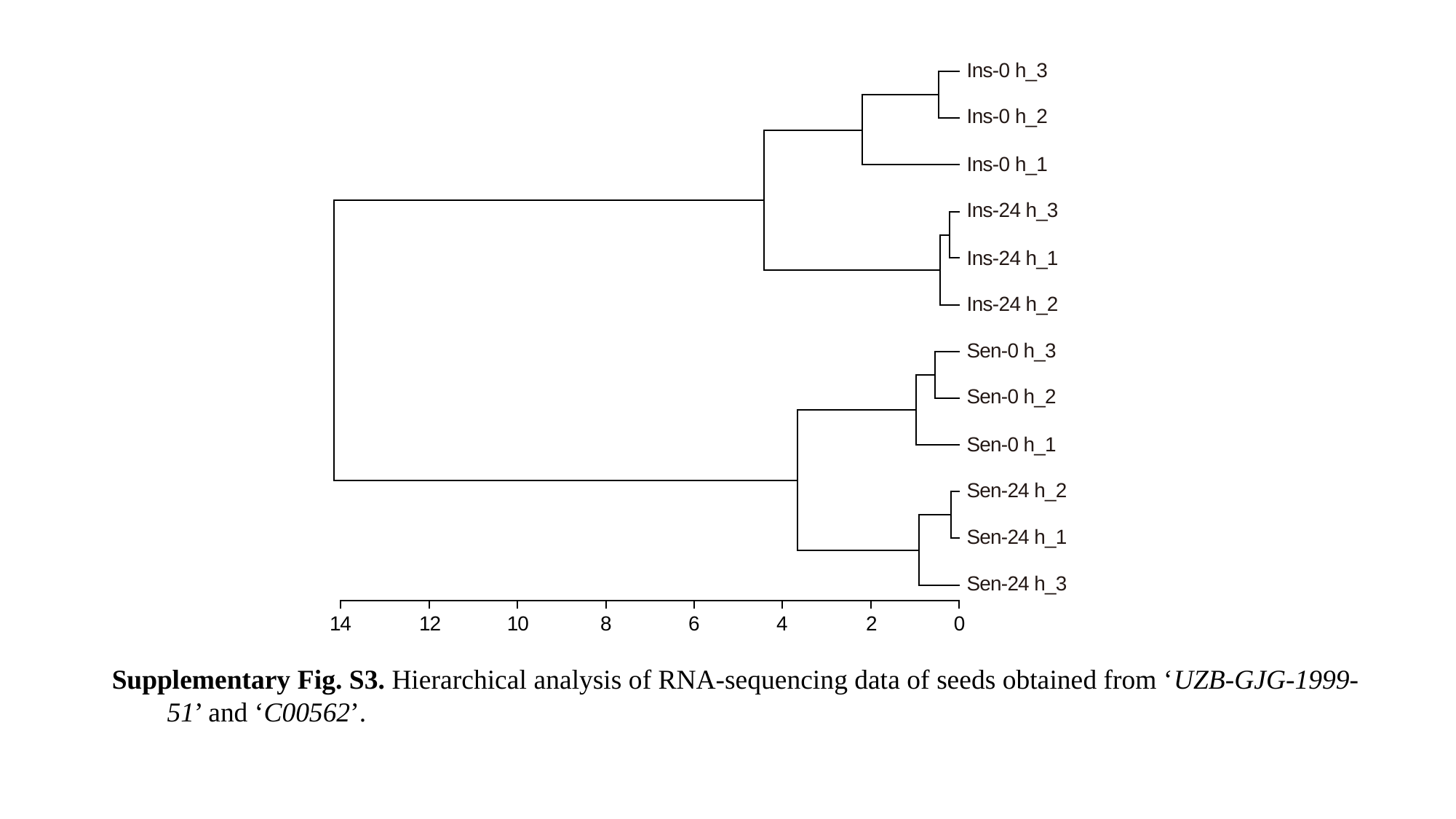

Supplementary Fig. S3. Hierarchical analysis of RNA-sequencing data of seeds obtained from ‘UZB-GJG-1999-
 51’ and ‘C00562’.

## Slide 7
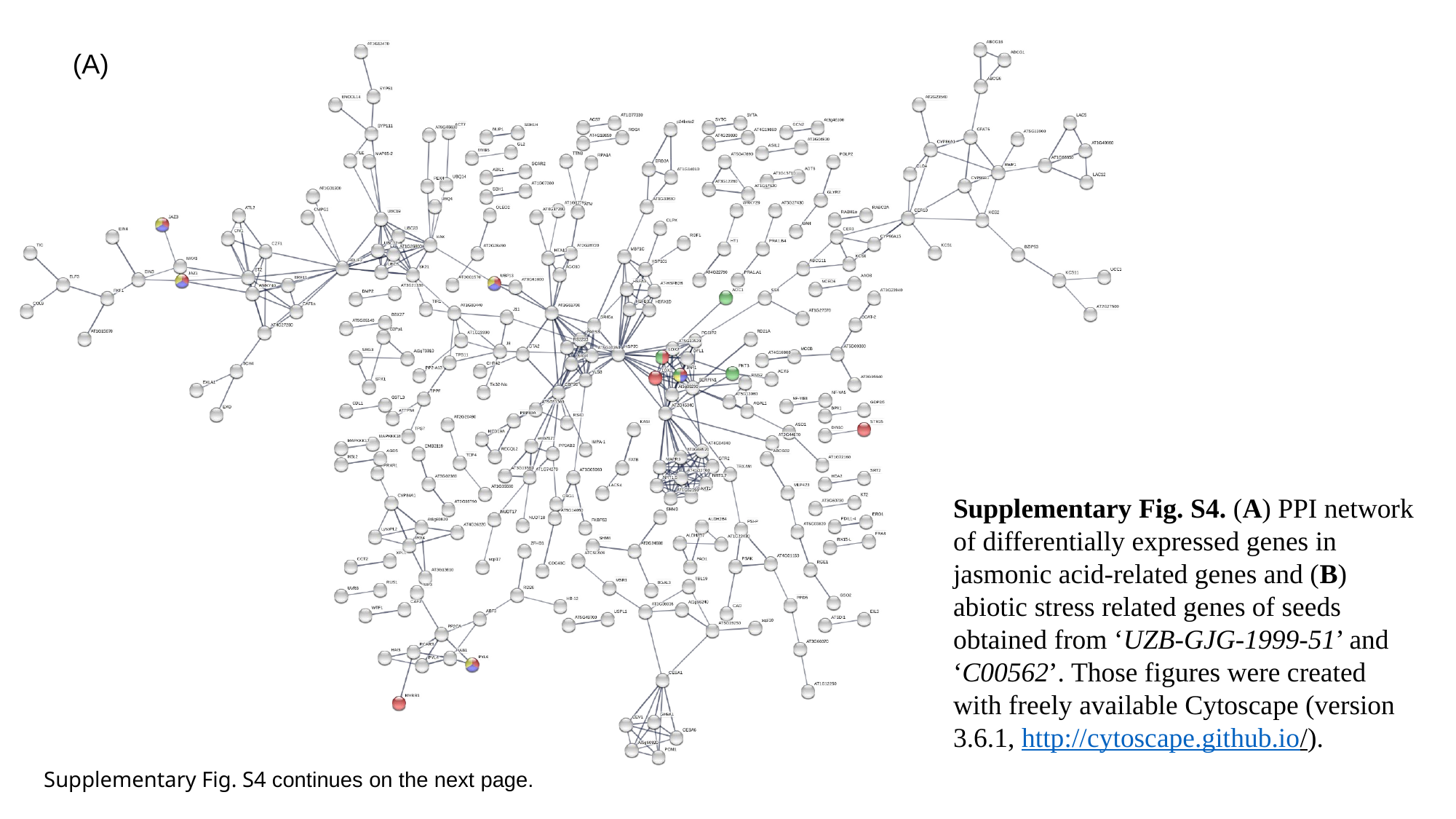

(A)
Supplementary Fig. S4. (A) PPI network of differentially expressed genes in jasmonic acid-related genes and (B) abiotic stress related genes of seeds obtained from ‘UZB-GJG-1999-51’ and ‘C00562’. Those figures were created with freely available Cytoscape (version 3.6.1, http://cytoscape.github.io/).
Supplementary Fig. S4 continues on the next page.

## Slide 8
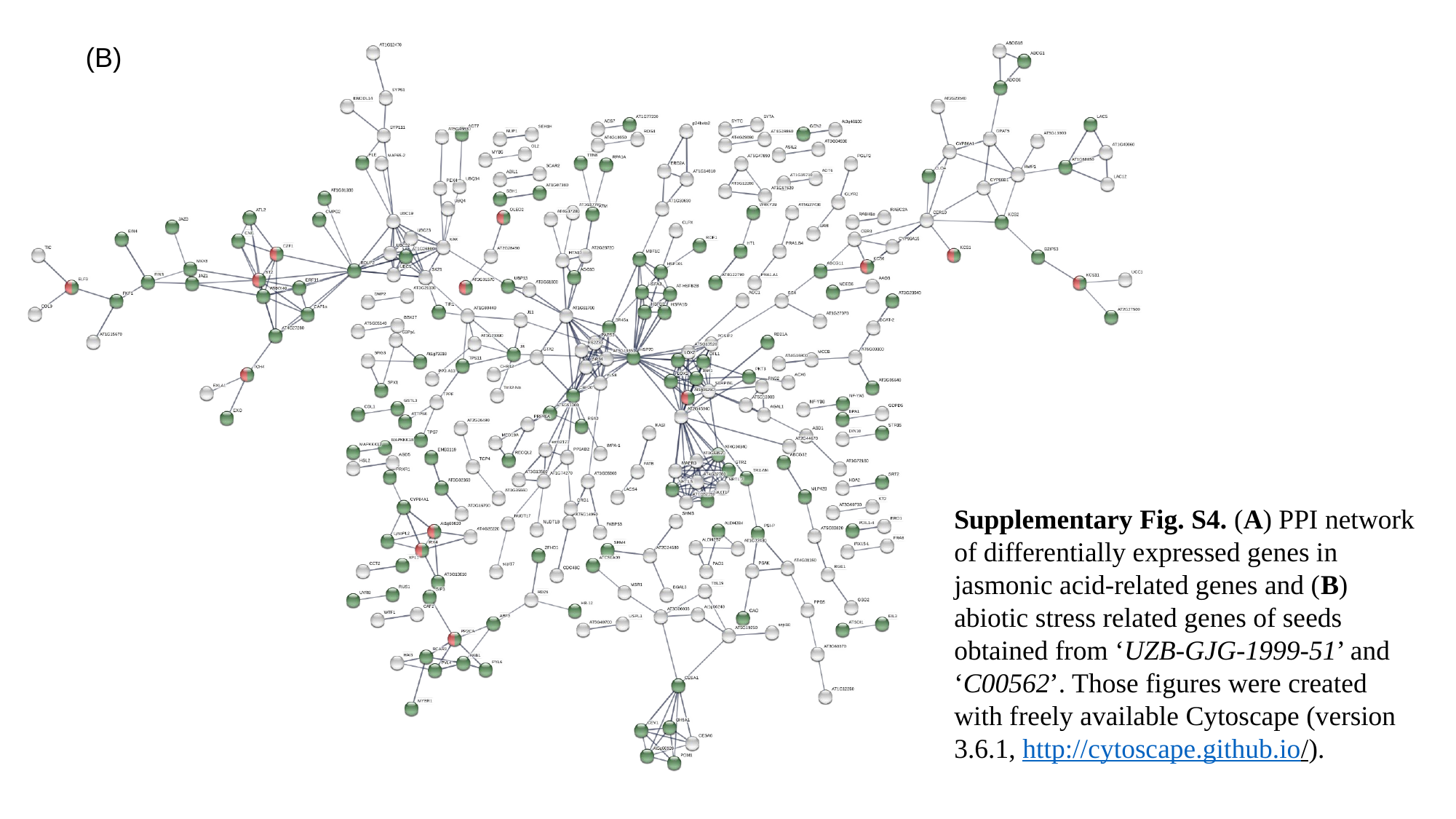

(B)
Supplementary Fig. S4. (A) PPI network of differentially expressed genes in jasmonic acid-related genes and (B) abiotic stress related genes of seeds obtained from ‘UZB-GJG-1999-51’ and ‘C00562’. Those figures were created with freely available Cytoscape (version 3.6.1, http://cytoscape.github.io/).

## Slide 9
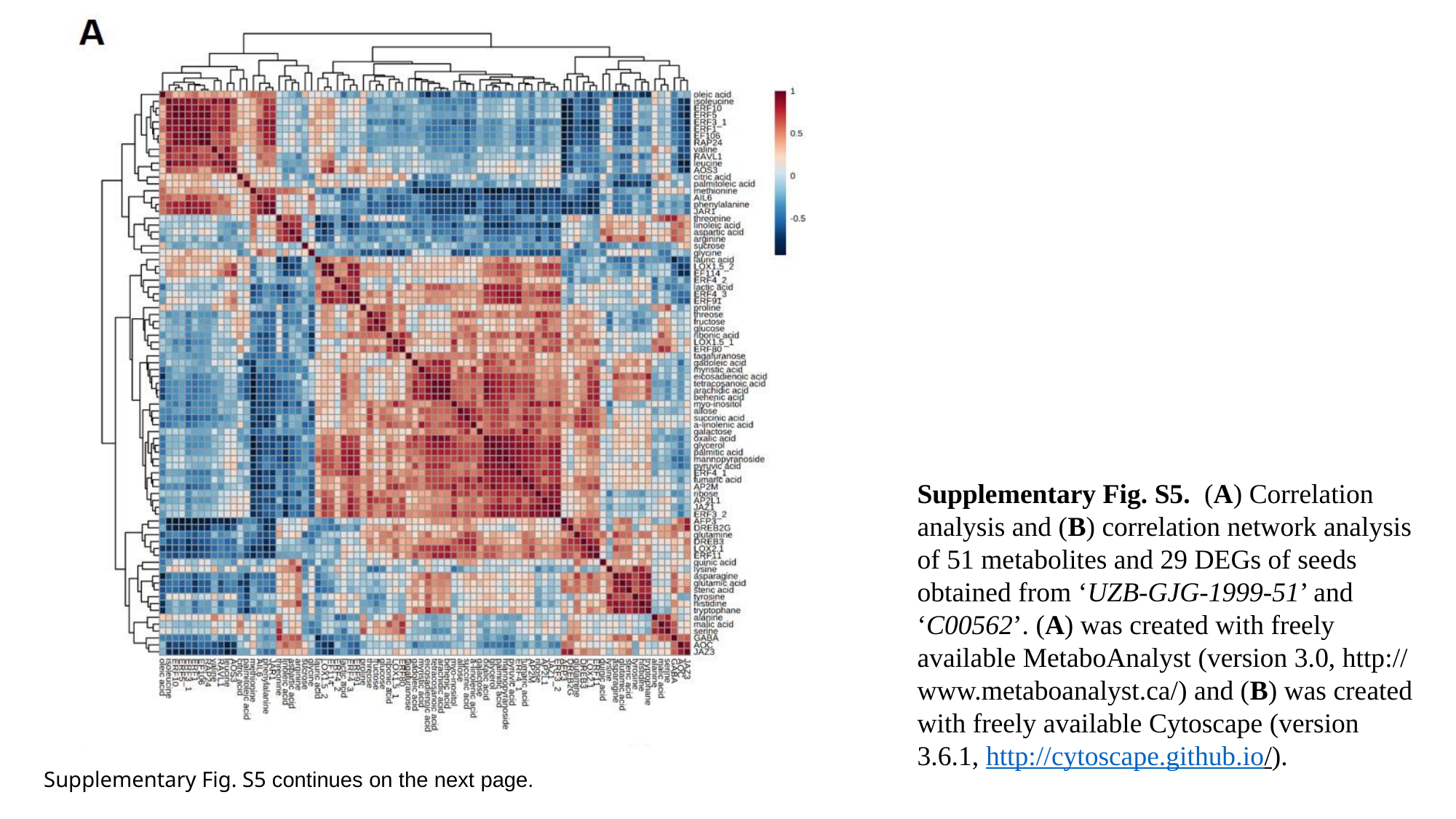

Supplementary Fig. S5. (A) Correlation analysis and (B) correlation network analysis of 51 metabolites and 29 DEGs of seeds obtained from ‘UZB-GJG-1999-51’ and ‘C00562’. (A) was created with freely available MetaboAnalyst (version 3.0, http://www.metaboanalyst.ca/) and (B) was created with freely available Cytoscape (version 3.6.1, http://cytoscape.github.io/).
Supplementary Fig. S5 continues on the next page.

## Slide 10
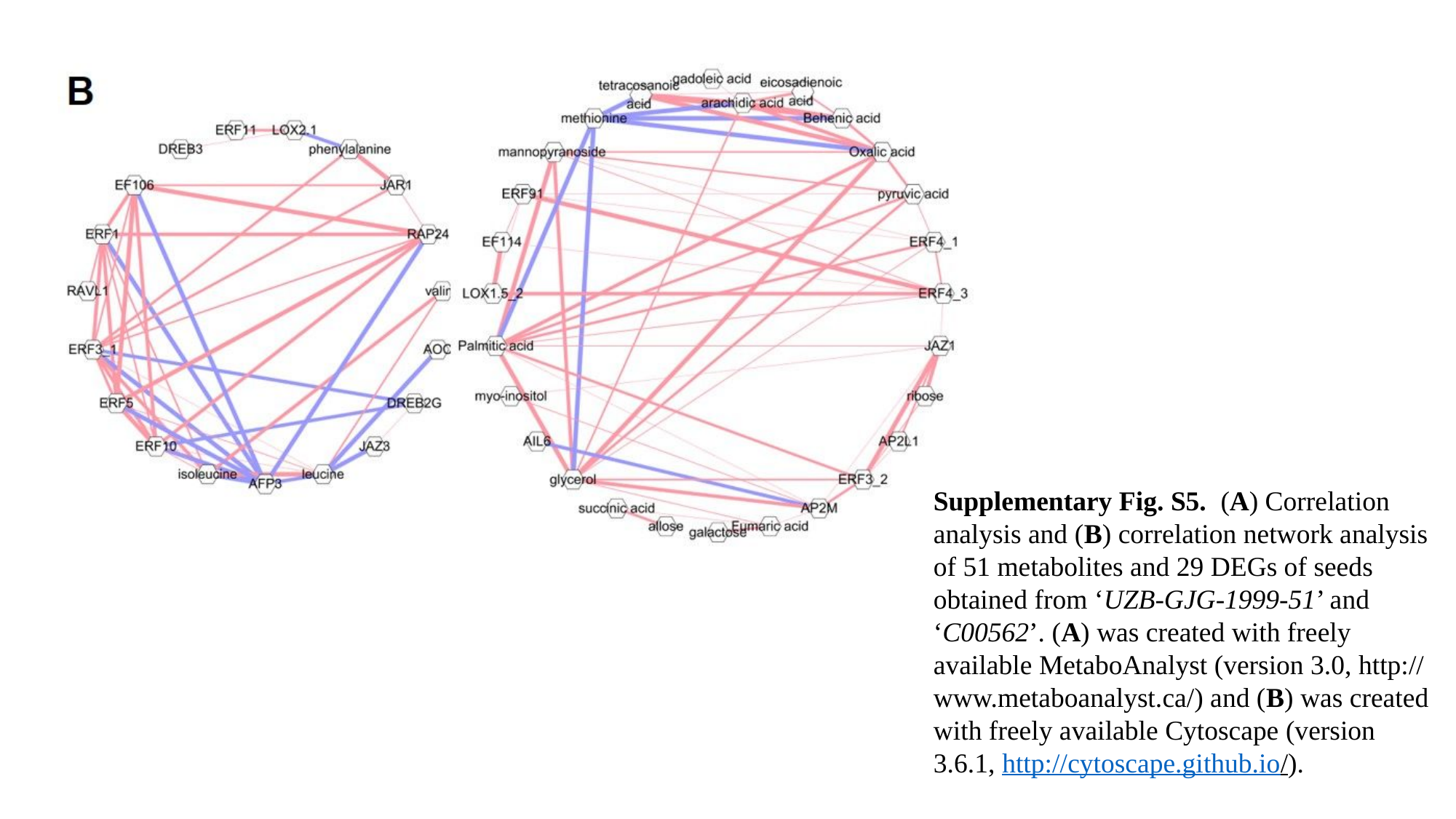

Supplementary Fig. S5. (A) Correlation analysis and (B) correlation network analysis of 51 metabolites and 29 DEGs of seeds obtained from ‘UZB-GJG-1999-51’ and ‘C00562’. (A) was created with freely available MetaboAnalyst (version 3.0, http://www.metaboanalyst.ca/) and (B) was created with freely available Cytoscape (version 3.6.1, http://cytoscape.github.io/).
