# Supplementary material for: Jasmonic acid and ERF family genes are involved in chilling sensitivity and seed browning of pepper fruit after harvest
Source: Sci Rep. 2020 Oct 21;10:17949. doi: 10.1038/s41598-020-75055-z (PMC7577993; doi:10.1038/s41598-020-75055-z)
Supplement: Supplementary file 2 — Supplementary Tables. [file 41598_2020_75055_MOESM2_ESM.docx]

**Supporting Information**

**_Tables_**

**Jasmonic acid and ERF family genes are involved in chilling sensitivity and seed browning of pepper fruit after harvest**

**Jeong Gu Lee ^1^, Gibum Yi ^1,2^, Jieun Seo ^1^, Byoung Cheol Kang ^1,2^, Jeong Hee Choi ^3^ and Eun Jin Lee ^1,4,^***

^1^Department of Agriculture, Forestry and Bioresources, College of Agriculture and Life Sciences, Seoul National University, Seoul 08826, Republic of Korea

^2^Plant Genomics and Breeding Institute, Seoul National University, Seoul 08826, Republic of Korea

^3^Korea Food Research Institute, Wanju-gun, Jeollabuk-do 55365, Republic of Korea

^4^ Research Institute of Agriculture and Life Sciences, Seoul National University, Seoul 08826, Republic of Korea

Jeong Gu Lee (tto12321@snu.ac.kr); Gibum Yi (gibumyi@gmail.com); Jieun Seo (grace0534@snu.ac.kr); Byoung Cheol Kang (bk54@snu.ac.kr); Jeong Hee Choi (choijh@kfri.re.kr); Eun Jin Lee (ejinlee3@snu.ac.kr)

*Correspondence to: ejinlee3@snu.ac.kr (E. J. Lee)

Tel.: (+82)-2-880-4565, Fax: (+82)-2-873-2056

**Supplementary Table S1**. 36 Genotypes of *Capsicum* *annuum* were screened according to the rates of seed browning induced by cold storage at 2° C for 3 weeks after harvest.

| **Accession name^1)^** | **Seed browning rate**  **(%)^2)^** |
| --- | --- |
| *C00562* | 63.86 |
| *MC 11* | 50.00 |
| *Sangju (Landrace)* | 48.98 |
| *Chili bangi* | 41.01 |
| *Jungangjongmyo-2000-6447* | 40.00 |
| *Chorbadjiyska* | 32.91 |
| *Gyeonggiyangpyeong* | 30.88 |
| *MK-4522* | 24.36 |
| *Wonsi-1984+pepper032506* | 24.00 |
| *MicroPep Yellow* | 18.52 |
| *Starburst_1* | 17.86 |
| *MC4* | 17.19 |
| *Filus blue* | 16.67 |
| *NPL-CGS-1986-22891* | 12.77 |
| *C01582* | 12.00 |
| *CV8* | 10.26 |
| *VC32* | 9.09 |
| *C00522* | 7.69 |
| *9425* | 5.36 |
| *9852-193 AVRDC 211* | 5.36 |
| *Hungarian Wax* | 4.31 |
| *Starburst_1* | 3.85 |
| *THA-Kasassert Univ.-15* | 2.70 |
| *CMV 980* | 2.44 |
| *HDA210* | 2.00 |
| *Super Hot* | 1.79 |
| *VP 84* | 1.33 |
| *UZB-GJG-1999-51* | 0.00 |
| *Thai Hot* | 0.00 |
| *C00590* | 0.00 |
| *Yolo Y* | 0.00 |
| *Ibam* | 0.00 |
| *CMV 1105* | 0.00 |
| *Takanotsume* | 0.00 |
| *Twilight* | 0.00 |
| *9456* | 0.00 |

^1)^The 36 pepper genotypes were obtained from the National Agrobiodiversity Center, Jeonju, Korea. Pepper seeds were planted in a greenhouse in Suwon, Korea. The plants were managed according to standard practices and pepper fruits were harvested at mature stages, approximately 45-50 days after full bloom, depending on genotypes. All pepper fruits were cold-stored at 2° C for 3 weeks under a relative humidity of 90 % and dark conditions.

^2)^The seed browning rate was observed after 3 weeks at 2° C and calculated using the following equation: Seed browning rate of a fruit (%) = number of browned seeds/number of total (normal + browned) seeds × 100. Twenty biological replicates of pepper fruit per each genotype were used to calculate the seed browning rate and an average rate of seed browning in each pepper genotype is presented.

**Supplementary Table S2.** Primers used for qRT-PCR analysis in the present study.

| **Gene** | **Forward primer sequence**  **(5′-3′)** | **Reverse primer sequence**  **(3′-5′)** | **Size (bp)** |
| --- | --- | --- | --- |
| *CaActin7* | TCTCCCCAACTACAAACAAC | CTGCTGTTTTAGGCAAGTCT | 160 |
| *CaERF1* | TTTAAATTTCCCGAGCCAGA | ATTTCTCAGCTACACCGTTT | 143 |
| *CaERF3_1* | CGATCAACTCTAGCTTCCAA | GTCTCAAATGTTCCTAGCCA | 143 |
| *CaERF5* | ACCGAGGTCCAAATATCATG | GGGATTGTGGAAATGCAAAA | 148 |
| *CaERF10* | TTCTTGTGGATGTTCGTGAT | CCAGAGTTGTTAGTTGGTGA | 141 |
| *CaERF11* | CCATTTGACAAGTCCATCTT | ATGCCAGTGGTAGAAGAAAA | 142 |
| *CaDREB2* | GATGATTCCGAGAGATTGAG | TAGCGGAAATAAGAGAACCA | 153 |
| *CaDREB3* | AGGTCACGTAGCCTTACTCA | AACAGGGCACTTCCATACTA | 146 |
| *CaLOX1.5_1* | AGCAAACAGTTTTCCCTACA | ACACCTTGTTCAGATTCAGG | 141 |
| *CaLOX1.5_2* | TGACCTGTTCTTCCATTTCT | GATGAGGTTTACTTGGGACA | 151 |
| *CaLOX2.1* | GAGGTGATGGAGTTGGAGTA | CTGCACCTTCTAGGATATGG | 155 |
| *CaAOC* | ATAACAGCAGGACTCTGCAT | CGAGGTAAGTGTCCTCGTAG | 150 |
| *CaAOS3* | GACGTTCCTTTTTCAGACAG | TGGAACTACTGATCCCAAAC | 145 |
| *CaJAR1* | CTTGGCTGTAGCAAAACACT | GAATCTTCCCTCATCAGACA | 148 |
| *CaJAZ1* | GTGTATTGTTGGCGTTGTTA | GAATCTGTTCCTTCAAGGTG | 140 |
| *CaJAZ3* | GGTGATGAACTTAGCACCAT | CGAGAGATCATTGCTAGGTC | 157 |
| *CaAFP3* | GCTAGGTGATCTTTCACTGG | TTCTGATTTTGGGAGTCAAC | 152 |

**Supplementary Table S3**. Summary of transcriptome sequencing data of seeds obtained from ‘*UZB-GJG-1999-51*’ and ‘*C00562*’.

| **Sample ID** | **Read No** | **Length** | **q30 (%)** | **q20 (%)** | **GC Ratio (%)** |
| --- | --- | --- | --- | --- | --- |
| Ins-0 h_1 | 25,759,330 | 3,889,658,830 | 96.54 | 98.07 | 44.34 |
| Ins-0 h_2 | 22,702,294 | 3,428,046,394 | 96.54 | 98.07 | 44.37 |
| Ins-0 h_3 | 27,138,742 | 4,097,950,042 | 96.48 | 98.03 | 44.28 |
| Ins-24 h_1 | 21,154,313 | 3,194,301,263 | 96.50 | 98.05 | 44.24 |
| Ins-24 h_2 | 23,984,330 | 3,621,633,830 | 96.51 | 98.05 | 44.31 |
| Ins-24 h_3 | 24,286,070 | 3,667,196,570 | 96.52 | 98.06 | 44.34 |
| Sen-0 h_1 | 19,602,840 | 2,960,028,840 | 96.59 | 98.09 | 44.36 |
| Sen-0 h_2 | 22,902,115 | 3,458,219,365 | 96.50 | 98.06 | 45.95 |
| Sen-0 h_3 | 16,987,530 | 2,565,117,030 | 96.62 | 98.11 | 44.18 |
| Sen-24 h_1 | 15,194,300 | 2,294,339,300 | 96.56 | 98.07 | 44.44 |
| Sen-24 h_2 | 33,344,577 | 5,035,031,127 | 96.56 | 98.08 | 44.63 |
| Sen-24 h_3 | 20,938,651 | 3,161,736,301 | 96.56 | 98.07 | 44.04 |

**Supplementary Table S4**. Differentially expressed gene lists in jasmonic acid-related genes and AP2/ERF family-related genes of seeds obtained from ‘*UZB-GJG-1999-51*’ and ‘*C00562*’.

| **SEQ-ID^1)^** | **FPKM^2)^ values** | | | | | **Description** |
| --- | --- | --- | --- | --- | --- | --- |
|  | **‘*UZB-JGJ-1999-51*’**  **(chilling**  **-insensitive)** | | | **‘*C00562*’  (chilling   -sensitive)** | |  |
|  | **0 h** | **24 h** | **0 h** | | **24 h** |  |
| GG5328 | 105.54 | 33.82 | 124.00 | | 44.07 | dehydration-responsive element-binding protein 2G (DREB2) |
| GG6586 | 9.94 | 1.70 | 29.71 | | 7.88 | dehydration-responsive element-binding protein 3 (DREB3) |
| GG8618 | 29.45 | 337.50 | 4.60 | | 130.36 | ethylene-responsive transcription factor 5-like (ERF5) |
| GG10682 | 40.17 | 152.86 | 12.99 | | 83.74 | ethylene-responsive transcription factor 3-like (ERF3_1) |
| GG13431 | 7.45 | 14.66 | 3.87 | | 6.09 | jasmonic acid-amido synthetase JAR1-like (JAR1) |
| GG14020 | 28.04 | 12.32 | 36.55 | | 56.06 | ethylene-responsive transcription factor 4-like (ERF4_1) |
| GG15208 | 9.10 | 1.04 | 9.53 | | 12.86 | ethylene-responsive transcription factor 80 (ERF80) |
| GG17684 | 0.68 | 1.91 | 4.52 | | 29.17 | ethylene-responsive transcription factor 91-like (ERF91) |
| GG18564 | 9.04 | 6.48 | 14.55 | | 14.95 | AP-2 complex subunit mu (AP2M) |
| GG22558 | 2.25 | 13.59 | 3.68 | | 7.80 | AP2/ERF and B3 domain-containing transcription factor RAV1 (RAVL1) |
| GG25022 | 7.39 | 8.26 | 15.24 | | 19.76 | ethylene-responsive transcription factor 3-like (ERF3_2) |
| GG32096 | 9.08 | 1.28 | 51.91 | | 16.93 | ethylene-responsive transcription factor 11 (ERF11) |
| GG32524 | 8.43 | 38.47 | 3.55 | | 18.54 | ethylene-responsive transcription factor 1-like (ERF1) |
| GG32959 | 9.77 | 0.98 | 27.72 | | 14.56 | probable linoleate 9S-lipoxygenase 5 (LOX1.5_1) |
| GG35399 | 80.68 | 47.12 | 40.96 | | 128.21 | ethylene-responsive transcription factor 4-like (ERF4_2) |
| GG36310 | 3.07 | 6.51 | 1.36 | | 1.39 | AP2-like ethylene-responsive transcription factor (AIL6) |
| GG41078 | 66.95 | 61.69 | 98.59 | | 246.98 | ethylene-responsive transcription factor 4-like (ERF4_3) |
| GG45556 | 65.61 | 161.46 | 110.95 | | 313.32 | ethylene-responsive transcription factor 114 (ERF114) |
| GG46889 | 31.48 | 11.44 | 19.92 | | 8.91 | allene oxide cyclase 4 (AOC) |
| GG49392 | 7.41 | 14.65 | 11.24 | | 34.03 | probable linoleate 9S-lipoxygenase 5 (LOX1.5_2) |
| GG50159 | 1.18 | 0.07 | 2.28 | | 3.99 | AP2-like ethylene-responsive transcription factor At1g16060 (AP2L1) |
| GG56978 | 7.59 | 1.99 | 26.17 | | 11.58 | linoleate 13S-lipoxygenase 2-1 (LOX2.1) |
| GG57571 | 11.15 | 8.24 | 33.36 | | 53.25 | protein TIFY 10A-like (JAZ1) |
| GG64653 | 62.12 | 118.27 | 33.89 | | 149.24 | allene oxide synthase 3 (AOS3) |
| GG66074 | 3.84 | 29.96 | 4.69 | | 19.19 | ethylene-responsive transcription factor 106 (ERF106) |
| GG68024 | 10.99 | 3.81 | 10.38 | | 2.99 | protein TIFY 6B-like (JAZ3) |
| GG71486 | 10.65 | 28.59 | 8.50 | | 21.59 | ethylene-responsive transcription factor 10 (ERF10) |
| GG76051 | 21.10 | 104.90 | 20.31 | | 50.45 | ethylene-responsive transcription factor RAP24 (RAP24) |
| GG80329 | 127.62 | 43.62 | 150.68 | | 84.81 | ninja-family protein AFP3 (AFP3) |

^1)^Differentially expressed genes with a false discovery rate < 0.05 were selected.
^2)^Fragment per kilobase of transcript per million mapped reads.
